# Versatility and Invariance in the Evolution of Homologous Heteromeric Interfaces

**DOI:** 10.1371/journal.pcbi.1002677

**Published:** 2012-08-30

**Authors:** Jessica Andreani, Guilhem Faure, Raphaël Guerois

**Affiliations:** 1 CEA, iBiTecS, Service de Bioenergetique Biologie Structurale et Mecanismes (SB2SM), Laboratoire de Biologie Structurale et Radiobiologie (LBSR), Gif sur Yvette, France; 2 CNRS, UMR 8221, Gif sur Yvette, France; 3 Université Paris Sud, UMR 8221, Orsay, France; MRC Laboratory of Molecular Biology, United Kingdom

## Abstract

Evolutionary pressures act on protein complex interfaces so that they preserve their complementarity. Nonetheless, the elementary interactions which compose the interface are highly versatile throughout evolution. Understanding and characterizing interface plasticity across evolution is a fundamental issue which could provide new insights into protein-protein interaction prediction. Using a database of 1,024 couples of close and remote heteromeric structural interologs, we studied protein-protein interactions from a structural and evolutionary point of view. We systematically and quantitatively analyzed the conservation of different types of interface contacts. Our study highlights astonishing plasticity regarding polar contacts at complex interfaces. It also reveals that up to a quarter of the residues switch out of the interface when comparing two homologous complexes. Despite such versatility, we identify two important interface descriptors which correlate with an increased conservation in the evolution of interfaces: apolar patches and contacts surrounding anchor residues. These observations hold true even when restricting the dataset to transiently formed complexes. We show that a combination of six features related either to sequence or to geometric properties of interfaces can be used to rank positions likely to share similar contacts between two interologs. Altogether, our analysis provides important tracks for extracting meaningful information from multiple sequence alignments of conserved binding partners and for discriminating near-native interfaces using evolutionary information.

## Author Summary

Unraveling how interfaces of protein complexes coevolved is of major importance to improve our ability to predict their structures and design novel binders. Proteins whose interaction was maintained throughout evolution generally have their homologs binding in a similar manner while their sequences can have significantly diverged. Constraints holding proteins together should be captured from the growing body of available multiple sequence alignments. However, it remains unclear which features of the interfaces provide most tolerance to mutations and it is unknown whether any invariant properties may help to extract meaningful signals from sequence alignments. To solve this issue, we tackled an unprecedented large scale analysis of more than 1000 non-redundant couples of structural interologs. Structural interologs are pairs of complexes of known structure whose chains are homologs. We quantitatively measured how the networks of contacts varied between two interfaces. Although highly versatile, we found that contact networks were more conserved for residues acting as anchors and for apolar contacts when they are clustered into surface patches. Altogether, our results provide major guidelines for exploiting the wealth of evolutionary information contained in the sequences of binding partners. On those bases we developed a method to predict which residues most likely conserve their contacts.

## Introduction

Protein-protein interactions are of fundamental importance in biological systems, and understanding the principles underlying these interactions is currently a major biological challenge [1], [2]. Two complementary sources of information about protein complexes are available. High throughput techniques deliver abundant information about protein-protein interaction networks. For every node of these networks, a number of homologous sequences can be aligned to highlight slowly evolving regions and pinpoint putative binding sites at the surface of proteins [3]. On the other hand, a smaller but significant and rapidly growing number of protein complex 3D structures provide high resolution data, available in the Protein Data Bank [4]. The general purpose of the present work is to explore the possibility of using the available structural information to improve our understanding and interpretation of sequence alignments. To combine these two approaches, we focused on the perspectives provided by evolutionary information. Indeed, in the course of evolution, multiple selective pressures occur at protein surfaces in order to preserve interactions between partners, so that protein interfaces are more constrained and evolve more slowly than the rest of the protein surface [5], [6]. However, these constraints are not specific enough to enable straightforward prediction of interfaces: in particular, most proteins have more than one possible interaction partner and their surface can contain several interface regions [7].

Building up on these evolutionary trends, the conservation of the global structure and architecture of complexes has been investigated. Above 30% sequence identity, the global quaternary structure of complexes was shown to be conserved [8], as was the binding mode for inter-molecular domain-domain interactions [9]. To capture the molecular principles determining common binding modes, there is a need for more detailed investigations of “interface structure conservation” [10]. This is precisely the approach that we adopt in the present study.

The evolutionary rate within the interface significantly depends on the degree of residue burial upon complexation [11]–[13]: evolution slows down in buried regions of the interface. Conserved residues also tend to be clustered in interfaces [14]. However, interface coevolution is a complex phenomenon. Correlated interface mutations are very difficult to pinpoint, in particular in transient interactions with an intrinsic need for fast adaptation [15], [16]. Identification of direct residue contacts from sequence alone through pairwise correlation analysis requires a large number of aligned sequences [17], [18]. Alternative methods exist to study more directly the coevolution of a given interface, but require a considerable amount of experimental effort [19]. The difficulty inherent in coevolution studies may be explained by the necessity to consider the local context of interface contacts [20], [21]. Relying on sequence analysis, the SCOTCH method revealed a great versatility in the way interface physicochemical complementarity is maintained across evolution and underlined the importance of local compensations [22]. Molecular aspects of evolution such as epistasis also revealed possible mechanisms for the observed variability in the way proteins achieve binding and interaction specificity [23], [24]. As mutual information is hard to extract from sequence alone, structure can be used as a complementary source of information to shed light on complex molecular coevolution events.

Protein-protein interactions rely on a global architecture which can be described at the geometric or the physico-chemical level. Complexes between proteins that have different global folds can share similar binding sites [25] and it appears that a limited number of protein interface architectures likely cover the whole range of cellular functions [26], [27]. The complementarity of interfaces is also based on a mosaic of physico-chemical properties at interface [28], [29]. Different binding strategies have been observed [30] and case studies illustrated the importance of salt bridges and hydrogen bond networks [31]–[33]. Medium scale studies showed that, although hydrophobic interactions are central to binding, especially in obligate interactions [34], some polar residues are also conserved [35].

Among these properties, it is still unclear which are the most relevant and how the underlying physico-chemical constraints can best be extracted from multiple sequence alignments. Such an issue is particularly critical when challenging a predicted docked model of complex against its evolutionary history. The in-depth structural analysis of homologous interfaces offers a unique opportunity to address this question in a quantitative manner. Our objective is to use as many structures of homologous interfaces as possible to understand the fate of deleterious mutations at the interface of complexes and to capture the most likely mechanisms buffering the destabilization of interfaces through the rewiring of contact networks. To tackle this challenge on a large scale, we relied on the InterEvol database [36] which we recently designed to explore the structure and evolution of protein complexes. In particular, InterEvol provides 1,024 non-redundant heteromeric structural interologs corresponding to conserved interactions between pairs of homologous protein chains [37]. We analyzed the conservation of interface contacts between these interologs, distinguishing between atomic, polar and apolar contacts. We found that overall, the conservation of polar contacts using usual descriptors is surprisingly low, rarely exceeding 30%. We thus explored whether alternative criteria may help to extract interface features correlated to a higher level of conservation. We show that anchor residues and apolar patches are of particular interest since they exhibit a significant increase in contact conservation. We also propose a hierarchy of interface properties which can be used to predict the likelihood for each residue to conserve its contact environment. These findings provide essential guidelines to read multiple sequence alignments and account for molecular plasticity at complex interfaces.

## Results

### From interface conservation to contact plasticity in interolog pairs

In order to analyze the conservation of interface contacts between pairs of residues, a large dataset of 1,024 pairs of non-redundant heteromeric structural interologs ( Dataset S1) was derived from the InterEvol database [36], going as far as possible in sequence divergence while retaining structurally similar binding modes (see section 15 in Text S1 and Figure S1 in Text S2). In particular, the interface root-mean square deviation between any two interologs is always below 8 Å and in most cases (82% of interolog couples) below 4 Å. The correlation between interface area within each couple of interologs is very good (correlation coefficient 0.98).

**Figure 1:**
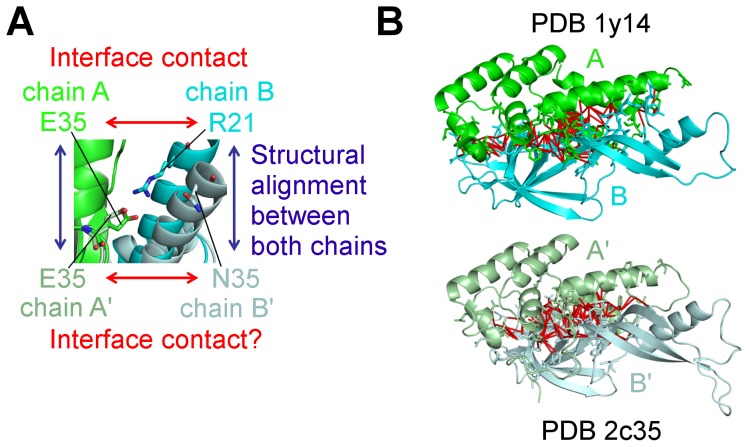
Definition of contact conservation between interologs. (A) Illustration of the way residue correspondence and contact conservation between interologs was assessed. (B) Illustration of atomic contacts at interface (red links), on the example of two interologous interfaces sharing 33% minimum interface sequence identity, between subunits of RNA polymerase 2 in yeast (upper complex, PDB id 1y14) and in human (lower complex, PDB id 2c35).

As a first step, we analyzed the global number of atomic contacts for each interface (see Methods and Figure 1B). We found that, on average, an interface contains 230 atomic contacts and the number of atomic contacts is roughly the same for any pair of interologs (the numbers differ by 15% on average between two interologs). Over an average of 61 atomic contacts when grouped into residue-residue contacts, each interface has on average 2 salt bridges, 9 hydrogen bonds (excluding backbone-backbone hydrogen bonds) and 35 apolar contacts (also grouped into residue-residue contacts). These distributions together with the composition of the interface and interface sub-regions is fully consistent with previous statistical analyses performed on smaller sets of heteromeric complexes [28], [29] (see section 1 of Text S1 and Table S1 in Text S2 for details).

**Figure 2:**
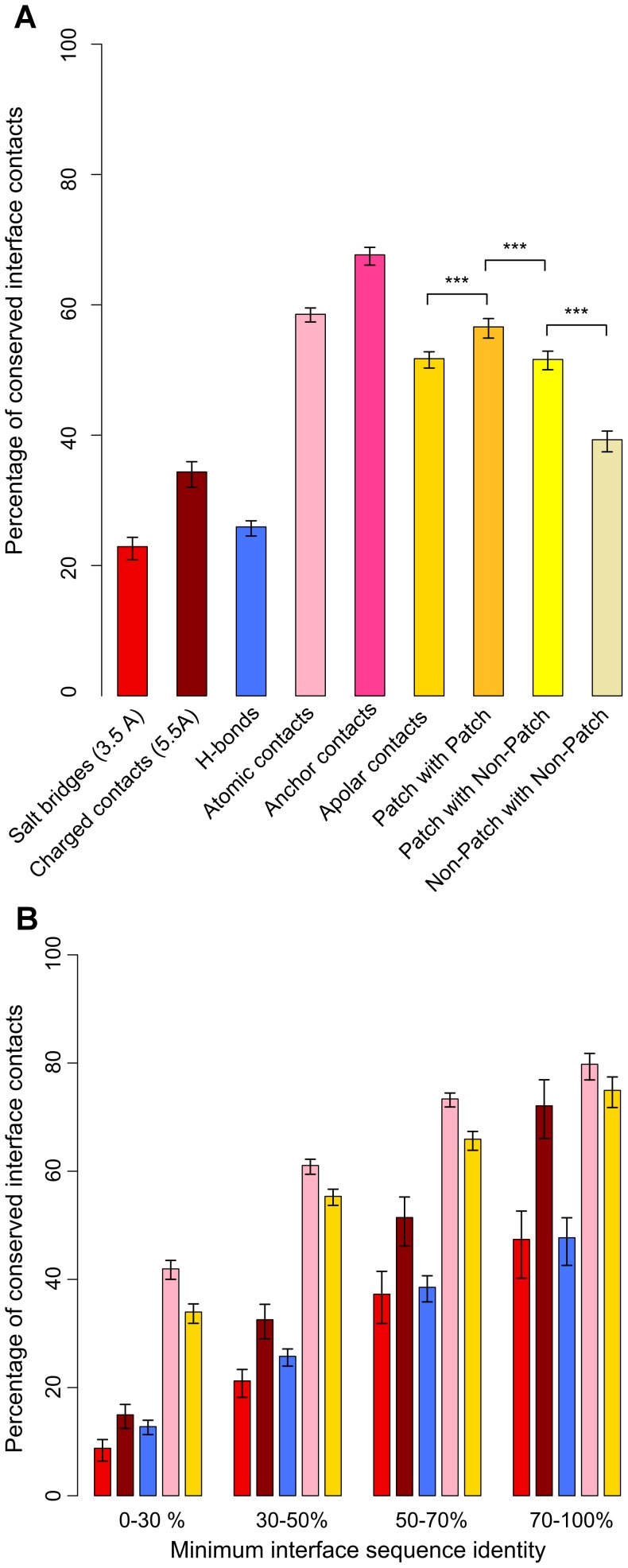
Versatility and invariance of different types of contacts. (A) Average contact conservation for all 1,024 pairs of interologs. From left to right, salt bridges at 3.5 Å (red), charged contacts at 5.5 Å (dark red), hydrogen bonds (blue), atomic contacts (pale pink), atomic contacts involving at least one anchor residue (dark pink), apolar contacts (gold), apolar contacts between residues both involved in apolar patches (dark yellow), apolar contacts between one residue involved in an apolar patch and one residue involved in no apolar patch (bright yellow), apolar contacts between two residues both non involved in apolar patches (light yellow). The mean of the contact conservation is plotted and the confidence intervals are based on a bootstrap procedure which consists in resampling half the database 1000 times, computing the mean conservation for the various categories of contacts, and representing the interval containing 95% of the calculated values. The p-values between distributions denoted by *** in this figure are <2e-5 in Wilcoxon rank sum tests. (B) Conservation among the different types of interface contacts for the four ranges of interface sequence identity. Colors are the same as in Figure 2A. For all types of contacts, the differences between any two distributions of conservation among the four groups of sequence identities are statistically significant (p-value<6.8e-3 in Wilcoxon rank sum tests).

We then compared the specific positions involved in each contact, weighting their contribution by the number of atomic contacts they were involved in. Corresponding residues between interologs were defined from a structural alignment, as illustrated in Figure 1A. It is important to keep in mind that the nature of the amino acid can vary between corresponding residues in two interologs (see Methods). Surprisingly, only 59.3% of contacts are conserved on average, meaning that between two homologous complexes, a given position in the interface of one monomer will likely interact with different positions in the binding partner. The corresponding mean of contact conservation is represented in light pink in Figure 2A. This corresponds to a range of situations depending on sequence divergence between the two interologs as displayed in Figure 2B. In order to have sufficiently populated subsets, the minimum interface sequence identities were binned into four categories, from very divergent to very similar interfaces: 0–30%, 30–50%, 50–70%, and 70–100%. A large spread in all distributions of contact conservation, represented in Figures S2A and S2B in Text S2, shows that the conservation of atomic contacts is heterogeneous among interologs. As expected, the higher the sequence identity at interface, the more contacts are conserved. In particular, contact conservation increases sharply between the very divergent interologs (below 30% sequence identity) and the less divergent interfaces.

The choice of looking at contacts to characterize plasticity implies to be careful about the different possible reasons for contact variation, which is why we defined several control datasets (details about the datasets are given in section 15 of Text S1, Dataset S1 and Figure S1 in Text S2). To ensure that the positions of side-chains are well defined, a higher-resolution dataset *interologs2.5* was constructed from the full dataset by restriction to X-ray structures with a resolution better than 2.5 Å. In *interologs2.5*, the average proportion of conserved contacts is 61.6%, very similar to the whole dataset. We also built a redundant, non-exhaustive dataset *redundant95*, containing 387 pairs of complexes with at least 95% overall sequence identity between both pairs of chains. In *redundant95*, we obtain on average 84.8% of conserved contacts. The results for *redundant95* are considered as an estimation of the experimental heterogeneity between near identical structures [38]. This heterogeneity explains part, but not all of the non-conserved contacts.

### Influence of conventional descriptors on contact conservation

Three sub-regions of the interface were defined depending on residue burial: core, support and rim (see Methods and Figure S3A in Text S2). A number of studies previously underscored the specificities of these sub-regions in terms of composition and evolutionary properties [11], [28], [39]. We found that atomic contacts in the core and support regions of the interface are significantly more conserved than the contacts in the rim region (see Figure S3B in Text S2). In particular, the atomic contact conservation for contacts involving at least one residue from the core and support regions (in any of the two interologs) is on average 63.2% while the atomic contact conservation for contacts involving at least one residue from the rim region (in any of the two interologs) is on average 48.9% (p-value<2.2e-16 using a non-parametric Wilcoxon rank sum test).

The individual evolutionary rates of interface residues were also found to modulate the conservation of the contacts they participate in: when the analysis is restricted to the most conserved residues in each interface (i.e. those with a normalized Rate4Site score [3] of more than 80, see Methods), the average contact conservation is 73.7%, significantly higher than the 59.3% average conservation over all residues (p-value<2.2e-16 using a non-parametric Wilcoxon rank sum test).

As could be expected, we thus identified the core and support regions as well as the slowly evolving positions as markers of contact conservation. However, various other conventional descriptors of the interface (types of secondary structure, obligate or non-obligate nature of the interaction, orthologous or paralogous relationship between interologs) were not found to influence significantly the conservation of atomic contacts (see section 2 of Text S1 and Figures S4A–B in Text S2).

### Switching out of the interface

When the non-conserved contacts are examined more closely, a striking proportion (around 39%) actually corresponds to cases where at least one of the two residues is no longer at interface in the interolog. This phenomenon, which we call “switching out of the interface”, is a major and non obvious cause of non-conservation.

In the whole dataset, on average, 26.4% of all interface residues “switch out” of the interface in the interolog. The percentage of “switching out” is lowered to 23.7% for *interologs2.5*. In *redundant95*, 11.4% of residues still switch out of the interface. Therefore, heterogeneity in the local structure of interfaces explains part, but not all of the fluctuations in the contours of the interface. Most fluctuations actually occur in the rim region: the “switching out” concerns mostly the interface periphery (see section 3 in Text S1 and Figures S4C–D in Text S2). Accordingly, 39% of rim residues switch out of the interface while 30% of support and only 7% of core residues switch out. Apart from the switching out effect, contacts involving rim residues are also intrinsically less conserved than for core and support residues. This double effect amplifies the versatility of contacts in the rim. Support residues switching out generally correspond to hydrophobic residues already quite buried in one monomer, which become even more buried in the interolog, to the point that they are no longer surface accessible. Looking in more details at the intriguing cases of core residues switching out, we observed that they generally correspond to residues which are further away from the geometric center of the interface or are not involved in a secondary structure element (see section 3 in Text S1 for further details).

To further investigate the relative contribution of different interface features to the probability for a residue to switch out, we used a logistic regression model. Details of the procedure are provided in the Methods. Briefly, from all the features tested in the logistic regression, six descriptors reported in Table 1 were found to significantly improve the prediction of residues switching out. These parameters come from two sources, relying either on sequence or on interface geometry. The logistic regression coefficients reported in Table 1 were estimated using a training dataset composed of the interface residues of one third of the 1,024 interolog couples selected at random. The other two thirds were used to test the predictive efficiency of the approach and the random splitting procedure was repeated ten times. For all the residues of the test dataset, the predicted probabilities to switch out were calculated. Residues were ranked and were progressively included in a ROC curve representing the fraction of true positives obtained versus the fraction of false positives. The resulting area under the curve reached 0.79 (Figure S5A in Text S2). The relative importance of the six features in Table 1 was analyzed by rating their impact for the reduction in the deviance of the logistic regression model. Detailed values are provided in Text S1 (section 14) and Table S2 in Text S2. Table 1 only summarizes that geometrical features are the most important, with the number of contacts of the residue and its location in the interface sub-regions (support, rim, core) as major contributors. When considering the structure of a complex and a sequence alignment of interologs, the equation provided in Text S1 (section 14) can thus be used to predict which residues are most likely to switch out from the interface.

**Table 1:** Contributions of the different interface features to the logistic regression models.

		Switching out			Contact conservation		
Category	Features	Coefficient in logistic regression equation	Significance	Rank in deviance reduction analysis	Coefficient in logistic regression equation	Significance	Rank in deviance reduction analysis
Sequence Features	Similarity residue (BLOSUM62)	−0.054±0.005	***	6	0.112±0.005)	***	3
	Similarity environment (BLOSUM62)	−0.128±0.005	***	3	0.224±0.007	***	1
	Overall sequence identity	−0.0096±0.0010	***	5	0.0066±0.0009	***	5
Geometric Features	Core/Support/Rim	0/0.96±0.08/1.06±0.07	***	2	0/0.23±0.04/−0.21±0.03	***	4
	Number of atomic contacts per residue	−0.122±0.006	***	1	0.075±0.002	***	2
	Distance to interface geometric center	1.40±0.09	***	4	−0.69±0.06	***	6
Intercept		−1.99±0.09			−1.32±0.06		

The 3 sequence descriptors are i) the similarity of the residue of interest with its structural equivalent in the interolog expressed as the substitution BLOSUM62 score, ii) the average of the substitution BLOSUM62 score for all the residues contacting the residue of interest (its “environment”), iii) the overall minimum sequence identity with the interolog (somehow correlated to the iRMSD). The 3 geometric descriptors are iv) the core/support/rim category of the residue of interest, v) the number of atomic contacts in which the residue of interest is involved, vi) the distance of the residue to the geometric center of the interface (normalized to a maximum of 1 for each interface). The logistic regression coefficients were averaged over the ten repeats. The interface category is a discrete feature and therefore its contribution will be 0 if the residue belongs to the core (default situation), 0.96 in the switching out predictor (resp. 0.23 in the contact conservation predictor) if the residue belongs to the support region and 1.06 (resp. −0.21) if the residue belongs to the rim. The standard deviations correspond to the variation of the coefficients over the ten repeats of the random partition of the residues into training and test datasets. The significance of each parameter was assessed from the z-tests performed on the logistic regression coefficients: significance values were always found to be <2.2e-16 (indicated by *** in the table). The parameters are ranked from the parameter contributing the most to the reduction in deviance (1) to the parameter contributing the least (6); this ranking is based on an analysis which consists in dropping each parameter one at a time from the logistic regression. Details can be found in section 14 in Text S1 and in Table S3 in Text S2.

### Versatility of polar contacts between interologs

We next analyzed the conservation of interface polar contacts: salt bridges and hydrogen bonds. On average, only 22.1% of salt bridges are conserved between interologs (Figure 2A, red bar). The spread in the distribution of salt bridge conservation for each pair of interfaces, represented in Figure S2A in Text S2 (red), is very high as there are few salt bridges per interface. The conservation of salt bridges is 23% in *interologs2.5* and 54.6% in *redundant95*. These values are surprisingly low for datasets of highly similar interfaces and high resolution: this is partly due to the restrictive distance cutoff (3.5 Å) applied when defining salt bridges. If we consider charged contacts with a distance threshold between charged atoms of 5.5 Å (dark red bar in Figure 2A), conservation reaches 86% in *redundant95*, while it remains below 35% on average in *interologs2.5*. This relaxed threshold was used in previous studies [11], [40] and provides a means to get indirect access to potentially water-mediated interactions, showing that such interactions could explain a much larger proportion of the missing salt bridges in *redundant95* than in the whole interolog dataset. Taken together, these results also show that experimental structural heterogeneity is largely insufficient to explain the very low conservation of salt bridges.

Figure 2B shows the mean values of polar contact conservation as a function of minimum interface sequence identity (in light red for salt bridges and dark red for longer distance charged contacts). Even at high sequence identity, the conservation of salt bridges is extremely low. However, the core and support regions and the evolutionary rate remain markers of higher salt bridge conservation. For instance, salt bridge conservation is 45.6% on average among residues with a normalized Rate4Site conservation score over 80, and this increase is significant for sequence identities above 30% (p-value<4.7e-3 in a Wilcoxon rank sum test). The distributions of polar contact conservation depending on interface sub-regions are represented in Figure S3E in Text S2.

Interestingly, cases of charge exchange between two positions (which are counted as conserved contacts) represent only 1% of the conservation cases, showing that binary substitutions of charged positions are extremely rare events in evolution. Therefore, artificial design strategies which search for direct effects of compensatory charge substitution to assess the physiological relevance of an interaction [41], [42] are not representative of events which happen in the course of evolution.

**Figure 3:**
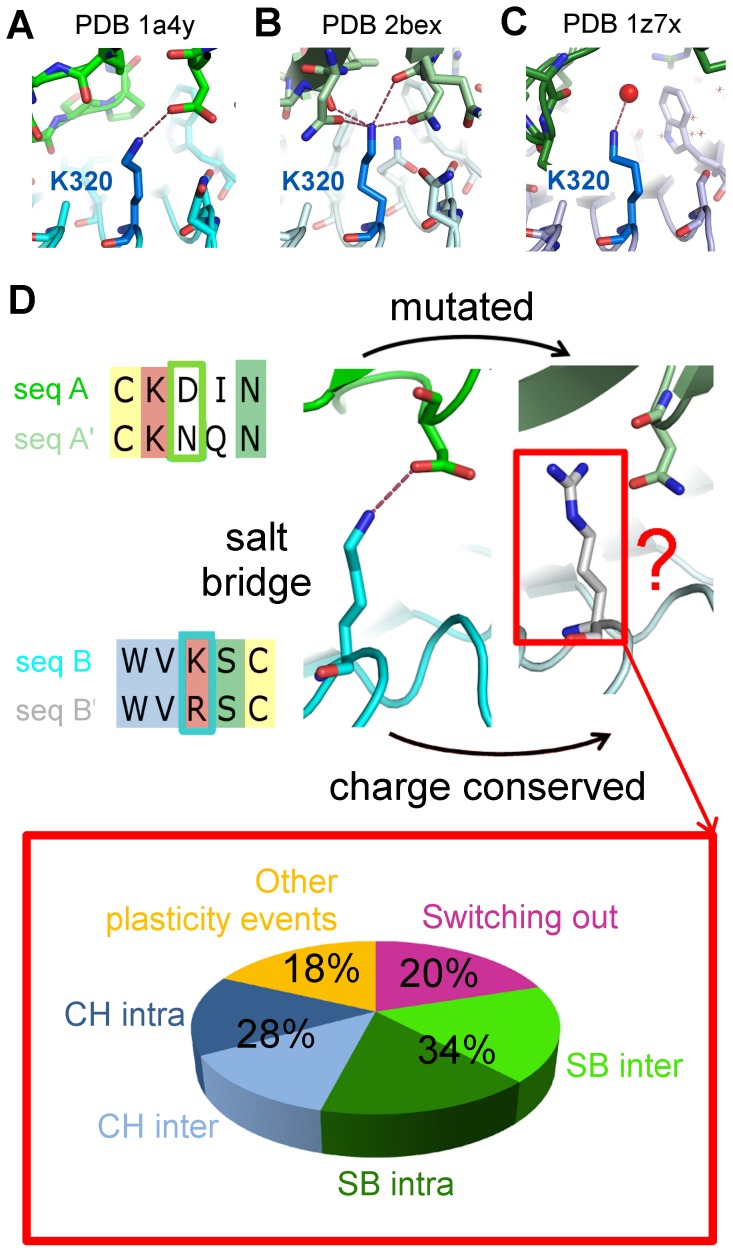
Scenarios of versatility in polar contacts. (A–C) The mutually interologous interfaces are interfaces in *Homo sapiens* between a conserved partner (ribonuclease inhibitor, in shades of blue) and three different partners with 30% to 38% mutual interface sequence identity (in green, light green and dark green; respective PDB ids of the complexes 1a4y, 2bex, 1z7x). The frames show a conserved lysine in the blue chains (K320 in chains A of 1a4y and 2bex and chain W of 1z7x) involved in three different environments: (A) an interface salt bridge, (B) a network of interface hydrogen bonds and (C) no interface contact but a hydrogen bond with interface water. (D) Fate of residues remaining charged but whose salt-bridge partner in the other interolog switches out of the interface or is mutated into an uncharged residue. 20% of the residues remaining charged actually switch out of the interface (purple), 34% remain involved in a salt bridge (either another salt bridge at interface, SB inter, light green, or an intra-molecular salt bridge, SB intra, dark green), 28% are involved in longer distance charged contacts (either inter-molecular, CH inter, light blue, or intra-molecular, CH intra, dark blue) and 18% (orange) correspond to other situations such as the involvement of the residue in hydrogen bonds or interactions with interface water molecules.

What happens in the cases of non-conserved salt bridges? 38% of non-conserved salt bridges correspond to at least one residue switching out of the interface, 15% correspond to a charged contact at longer range in the interolog and 40% correspond to the mutation of at least one of the two residues into an uncharged residue. In interfaces where salt bridges are lost, other intra- or inter-molecular salt bridges or charged contacts generally occur so that charged residues do not remain isolated. In 75% of the situations where one of the two residues is mutated into a neutral or opposite-charge residue or switches out of the interface, but the other remains charged and at interface, the latter residue stays involved in at least one intra- or inter-molecular salt bridge or charged contact. This means that although the residues involved in salt bridges are frequently mutated, there remain significant charge compensation constraints and the energetic frustration created by a lost salt bridge or charged contact seems to be easily released through local plasticity, as illustrated in Figure 3A–C. The type of plasticity events expected for charged residues in an interface can be quantified from this analysis (see Figure 3D and Figure S6 in Text S2), which provides guidelines in interpreting their substitutions in multiple sequence alignments.

The conservation of hydrogen bonds is also low: 27.8% on average for the whole dataset (blue bars in Figure 2A–B). Hydrogen bond determination is particularly sensitive to resolution as it relies on precise geometry and orientation of atoms. We thus checked if the conservation was still low in a context of good resolution and high redundancy. The conservation is 62.4% (respectively 65.6%) in the subsets of *redundant95* with resolution better than 2.5 Å (respectively 2.0 Å), 29.7% in *interologs2.5* and 30.0% in the subset of interologs with resolution better than 2.0 Å. This shows that hydrogen bonds are extremely versatile (see sections 5 and 6 of Text S1 and Figure S4C–D in Text S2 for a detailed analysis of the cases of non-conserved hydrogen bonds). Overall, plasticity in hydrogen bonds occurs up to high sequence identities, although it is not as pronounced as for salt bridges. However, there are strong constraints on each interface so that most polar residues satisfy their hydrogen bonding potential, as detailed in section 6 of Text S1.

### Apolar contacts are conserved between interologs thanks to apolar patches

Apolar contacts were calculated as all atomic contacts involving apolar surface atoms (C or S) from any type of interface residue (see Methods). On average, 51.7% of interface apolar contacts are conserved between interologs, as displayed in golden yellow in Figure 2A. The apolar contact conservation is 54.9% in *interologs2.5* and 80.8% in *redundant95*. A significant part of the non-conservation is thus due to the heterogeneity in the local structure of the interface. In Figure 2B, the average apolar contact conservation (in golden yellow) as a function of minimum sequence identity at interface clearly shows that apolar contacts are much more conserved than polar contacts for any range of interface sequence identity.

Non-conserved apolar contacts correspond to at least one residue switching out of the interface in 25% of all non-conservation cases. More interestingly, in 48% of the non-conservation cases, the residues are no longer in pairwise apolar contact, but contact can be recovered through their involvement in apolar regions of the interface called apolar patches.

**Figure 4:**
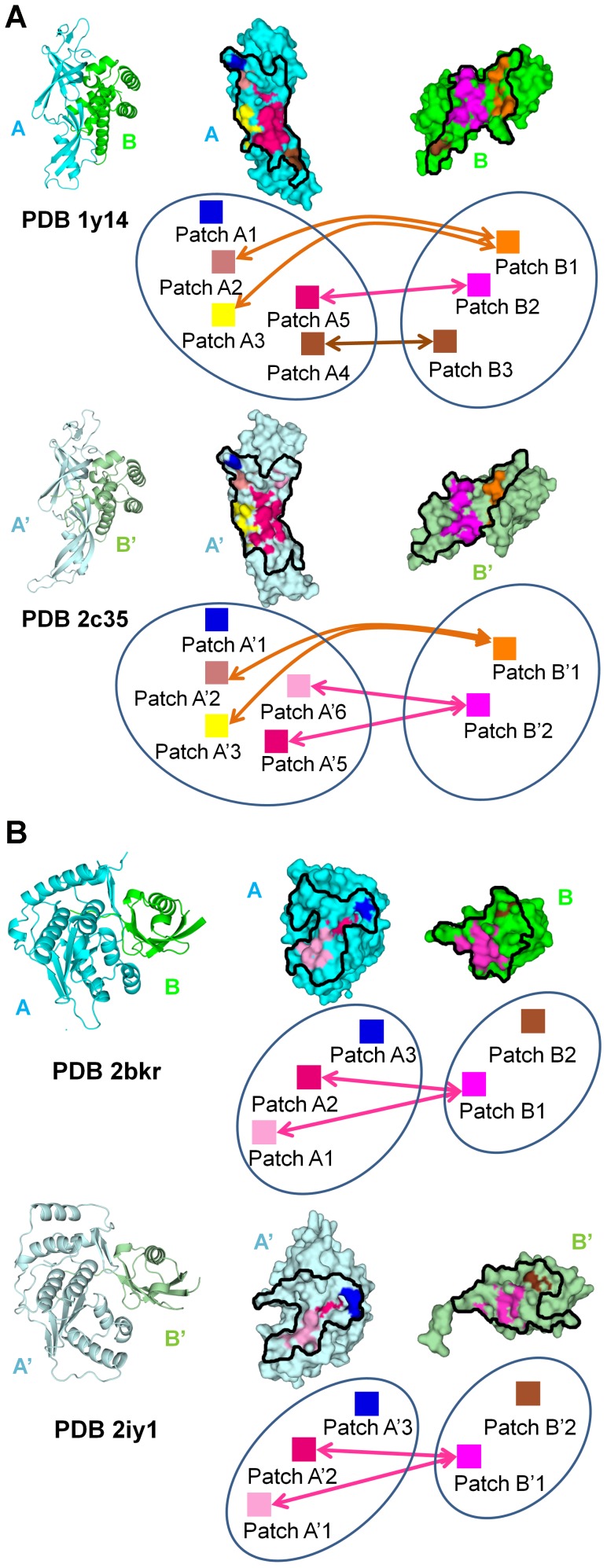
Apolar patches for two pairs of interologs. In each pair of interologs, the interfaces are split open. The contours of the interface on each chain are delimited by black lines. Patches of the same color in both interologs represent equivalent patches between homologous chains. Patches of identical or similar color on each side of the interface represent patches in contact at interface. For each chain, a diagram is shown with labels describing the relationship between patches (for instance, Patch A1 and Patch A'1 are structurally aligned between interologs) and arrows connecting the apolar patches in contact. (A) Two interologous interfaces sharing 33%; minimum interface sequence identity, between subunits of RNA polymerase 2 in yeast (upper complex, PDB id 1y14) and in human (lower complex, PDB id 2c35); in this case, contact conservation between patches is 84%. (B) Two interologous transient interfaces in human sharing 25% minimum interface sequence identity, NEDD8/NDP1 (upper complex, PDB id 2bkr) and SUMO1/SENP1 (lower complex, PDB id 2iy1); in this case, contact conservation between patches is 100%.

Hydrophobic patches were previously characterized on protein surfaces and protein-protein interfaces [43], [44] and shown to be meaningful in the perspective of prediction or protein design [45]. Here, apolar patches are identified as contiguous regions of the interface connected through at least four interface atoms from at least two different interface residues and sharing a delocalized property of apolarity (see Figure S7A in Text S2). Among the interfaces of the whole interolog dataset, on average 41% of all interface residues are involved in an apolar patch through at least one of their atoms. Apolar patches are well conserved between interologs, as illustrated in Figure 4. 82% of patches correspond to an equivalent patch in the interolog and fluctuations in the patches occur mostly at the periphery of the patch.

We first distinguished the behavior of residue pairwise apolar contacts with respect to their location in an apolar patch. We considered 3 situations, depending whether i) both residues in contact are in a patch, ii) only one is in a patch, iii) none of them is in a patch. There is a significant difference in the conservation trend between the three, supporting the view that apolar patches coincide with a location in which apolar contacts are more conserved (Figure 2A, three bars on the far right in shades of yellow). In particular, apolar contacts between two residues involved in apolar patches (mean conservation of 57%) are much more conserved than apolar contacts between two residues not involved in apolar patches (mean conservation of 39%) (p-value<2.2e-16 in a Wilcoxon rank sum test).

Rather than looking at apolar contacts between two specific residues, we also considered the possibility that apolar patches may conserve their contacts at the patch level, irrespective of the specific residues involved in the contacts. Under these conditions, we measured that the average conservation of the apolar contacts when clustered into bundles of contacts between two apolar patches reaches 84%. This high level of contact conservation holds for the different ranges of sequence identity (see Figure S7B in Text S2). The level of conservation of apolar contacts between patches is not directly comparable to atomic contacts since they involve clusters of residues, which makes them inherently more robust to mutations. To better assess the significance of such an increased conservation, we further probed what would be the conservation of apolar contacts between two randomly selected patches in contact. Random patches were generated with a distribution of patch size and distributions within core, support and rim as close as possible to the distributions for the real apolar patches (see detailed procedure in Text S1, section 10). We also controlled that random patches faced each other in a manner similar to that occurring between naturally observed apolar ones. The average conservation of apolar contacts when they are considered between random patches (clusters of residues) rather than at the residue level ranges from 58% to 63% depending on the level of constraint that we applied to define the random patches. Whatever the conditions these values are significantly below (p-value<2.2e-16 in Wilcoxon rank sum tests) the average conservation of 84% observed for real apolar patches (Figures S7D and S7E in Text S2).

Therefore, although apolar contacts appear moderately conserved at the pairwise residue level, apolar patches have a reservoir of plasticity for the contacts they engage in which can buffer the way contacts are rewired during evolution. For two apolar patches in contact, on average 65% of the residues involved in the patch participate actively in the contact and this proportion is similar in the two interologs.

We wondered whether the property of apolar contact conservation between patches was mainly observed for obligate complexes and whether it would hold true if we consider the most transient interactions contained in the dataset. From a close inspection of the functions associated with the non-obligate interfaces, we extracted a non-exhaustive subset of 60 pairs of interologs that we could confidently assign to the transient category (flagged in Dataset S1, see section 15 in Text S1 for details). This subset was sufficient to consider meaningful statistics. Remarkably, we found that the distribution of apolar contact conservation between apolar patches obtained for this subset of interactions was undistinguishable from the rest of the dataset (Figure S7C in Text S2).

### Conservation of anchor residues and their interface contacts

“Anchor residues” were initially identified as the surface residues burying the largest solvent-accessible surface area upon binding (usually one residue with ΔrASA≥100 Å^2^, but sometimes two or three residues with a slightly lower ΔrASA) and shown to generally coincide with functionally or kinetically important positions or energetic hot spots [46]. Compared to hot spots, the notion of anchor has the advantage that it can be defined on a geometric basis without thermodynamic analysis. Interface anchoring was also previously used in design [47], [48].

For each interface in the interolog dataset, we identified the three residues from the core and support regions that bury the most accessible surface area upon binding (and in any case, over 80 Å^2^) as “anchor residues”. Details about anchor residues are provided in Text S1 (sections 11 and 12) and Figure S8 in Text S2. In particular, anchor residues make more interface contacts than other core residues: on average an anchor residue makes 24 atomic contacts (with a standard deviation of 10) and other core residues make 10 atomic contacts (with a standard deviation of 7). The distributions of the number of atomic contacts are significantly different between anchor residues and other core residues (p-value<2.2e-16 in a Wilcoxon rank sum test). The proportion of anchor residues which are aligned with another anchor residue in the interolog is on average 53%. As a control, in *redundant95*, 74% of anchor residues are aligned with another anchor residue in the interolog.

**Figure 5:**
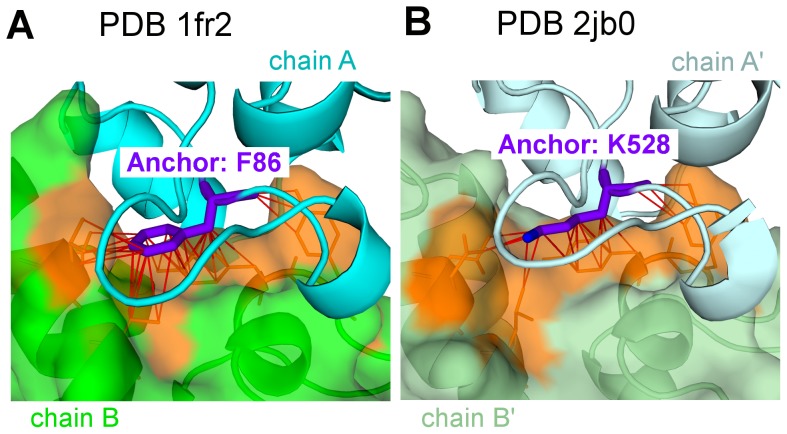
Conservation of the anchor position and its atomic contacts in colicin/IM complexes. The represented interfaces are colicin E9 (green) in complex with IM 9 (cyan) (PDB id 1fr2) and colicin E7 (pale green) in complex with IM 7 (pale cyan) (PDB id 2jb0). The Phe in 1fr2 (F86, chain B) and the structurally aligned Lys in 2jb0 (K528, chain B) are both anchors (highlighted in purple) and the conservation of atomic contacts involving this position is 76%. The residues in orange are those contacting the anchor residues.

The conservation of contacts involving at least one anchor residue was assessed. The average atomic contact conservation is raised from 63.2% for contacts involving core and support residues to 67.7% for contacts involving anchor residues (p-value = 3.7e-8 in a Wilcoxon rank sum test). This is illustrated in Figures S8E and S8F in Text S2 and by the dark pink bar in Figure 2. The conservation of anchor neighbors and atomic contacts is illustrated in Figure 5 in the case of an anchor which conserves 76% of its contacts although the substitution of a phenylalanine by a lysine residue represents a drastic physico-chemical change. The average conservation of contacts involving at least one anchor residue whose structural equivalent in the interolog is also an anchor residue is raised to 83%. Therefore, in comparison to the properties of the core, the relative increase in anchor conservation is moderate but significant. The general trend is globally that the more contacts a residue make across an interface, the more its contacts tend to be conserved. This is true in particular for anchor residues, which concentrate many interface contacts. This is consistent with the results we observed in developing the contact conservation predictor discussed below.

### Can conservation of contacts be predicted despite interface versatility?

The large scale analysis of interolog structures reveals a significant versatility in the way contacts redistribute among interfaces. We wondered whether several of the features that were analyzed might be combined to improve the prediction of the residues likely to conserve their contacts. We explored two distinct strategies either using the information contained in the set of redundant complexes from the *redundant95* or following the logistic regression approach as developed before in the switching out section.

First, we asked whether the structural flexibility observed in the set of *redundant95* complexes might correlate with residues likely to change their contact networks during evolution. This idea follows the observations in [49] where the authors observed an overall relation between protein dynamics and conformational changes enabling sequence changes in evolution. From the InterEvol database, we could extract 52 cases of interfaces where for a given complex, we had the structures of both a redundant complex (with over 95% sequence identity) and at least one structural interolog. Relying on these 52 cases (listed in Table S3 in Text S2), we compared the structural flexibility (contact conservation between a complex of interest and its redundant complex) and the evolutionary flexibility (contact conservation between the same complex of interest and its interolog(s)). Concerning salt bridges for example, we found that when a salt bridge is conserved in the redundant complex, then it is also present in the interolog in 35% of cases, a percentage significantly higher than the average 22% observed over the whole database. More details are provided in Text S1 (section 13) for salt bridges and apolar contacts. Overall, the conservation of contacts in the redundant complexes provides some information about the potential conservation of contacts in the interolog.

The second predictive approach aimed at developing a logistic regression model following the same procedure as in the switching out section. We searched which parameters would best discriminate the residues likely to retain most of their contacts between two interologous interfaces. A third of the 1,024 couples of interologs was randomly selected and used as the training dataset for the estimation of the logistic regression coefficients. The same six features as those identified in the switching out section were actually found to best predict the contact conservation of interface residues. The hierarchy of their importance is however different as shown by the impact of each of the six features on the reduction in deviance (see Table 1 and details in section 14 of Text S1). The sequence similarity scores of the residues contacting a residue of interest (its “environment”) now appears as the predominant variable followed by the number of contacts a residue is involved in. In contrast, the contribution of the different sub-region categories (core/support/rim) appears less prominent. From the coefficients reported in Table 1, it is possible to use the equation in Text S1 (section 14) to predict the residues most likely to conserve their contacts. When testing the predictive power of the logistic regression on the test dataset (the remaining two thirds of the interolog couples), the area under the ROC curve reached 0.75 (Figure S5B in Text S2).

It is particularly interesting that the number of contacts in which a residue is involved plays such a significant role since it is directly related to our observations that many anchors exhibit significant contact conservation with respect to other residues of the core. The property of anchors can be seen as the salient feature of the more generic “number of contacts” descriptor. Accordingly, there is also a significant enrichment of anchor residues among the best residues predicted from the logistic regression model supporting their ability to concentrate a conserved network of contacts (see section 14 in Text S1).

## Discussion

In this study, we have observed that although the binding modes between structural interologs are globally well conserved, there is a great diversity in the details of the interface arrangements at the residue level. Among the variety of the properties analyzed through the 1,024 couples of interologs, contact conservation was found significantly increased in two structural contexts: (i) between apolar patches and (ii) around anchor residues. These notions are defined based on the geometry and composition of each interface, independently of the concept of structural interology. We show that if we restrict the analysis to a few residues per interface which are actively involved in the binding (anchor residues), contact conservation is significantly higher than for core and support residues (Figure S8 in Text S2). Even more strikingly, apolar patches are maintained between interologs and apolar contacts between these patches are especially well conserved. This is critically important when interpreting multiple sequence alignments under the assumption that a set of positions maintained their mutual contacts throughout evolution. Anchors can change their physico-chemical status as illustrated in Figure 5 (the distribution of anchor amino acid types is given in Table S4 in Text S2) while apolar patches maintain their hydrophobic character distributed over a number of positions. It is important to underscore that non-hydrophobic residues also have to be considered to build these clusters, revealing such an invariant property. Hydrophobic residues represent 71% of the contributors to the apolar patches and significant contribution is also brought by arginine and to a lesser extent lysine side-chains (see Table S4 in Text S2). The conservation of both anchors and apolar patches holds true for obligate and non-obligate interfaces as predicted by NOXclass [50]. Among the non-obligate complexes, we isolated a subset of 60 pairs that we confidently assigned to the class of transient interactions and found that the conservation properties were the same, underscoring the robustness of our findings to multiple types of interfaces (Figures S2A and S7C in Text S2).

We observed that polar contacts are much more versatile than apolar contacts, both in obligate and non-obligate interfaces. This is particularly intriguing since every interface satisfies its constraints on charge compensation (although sometimes at longer distances) and hydrogen bond saturation. In particular, salt bridges are very versatile, in agreement with a recent analysis of charged residue pairs performed on another set of homologous interactions [51]. Swapped charges almost never occur and strict conservation of charged residue pairs is rather rare. We go several steps further in tracking the fate of positions with apparent non-conserved salt-bridges or polar contacts. For every scenario of non-conservation, such as the loss of one charged residue in a salt-bridge pair or a mutation from a polar to an apolar residue, we quantified the mechanisms by which favorable interactions may be recovered (Figure 3, sections 6 and 7 of Text S1 and Figure S6 in Text S2), laying the basis for a probabilistic framework on how to infer the type of plasticity events which may occur depending on the sequence divergence.

Our systematic study thus opens several key perspectives regarding the mechanisms of interface evolution for heteromeric complexes. So far evolution of homo-oligomers has been particularly scrutinized underscoring the importance of symmetry [8], [52], [53], the relative simplicity of tuning oligomeric states by introducing large hydrophobic amino acids at interfaces [54] and the role of insertions and deletions in enabling or disabling specific binding modes [55]. Here, we have more specifically investigated the plasticity at the position and side-chain levels in hetero-oligomers. The high degree of interface plasticity is probably tolerated through conformational epistasis allowing for mutations with little consequence to accumulate and change locally the environment until more drastic changes occur [24]. In this evolutionary scheme, our observations suggest that although epistatic events can significantly induce sequence drifts, apolar patches in contact do not dissolve during the evolution of homologous interfaces. While conservation of apolar patches may ensure a basal affinity between members of two protein families, variation in the nature of the anchors may switch binding specificities as a first order effect. Such an effect was observed and experimentally challenged [23], [56] in the case of the colicin-IM complex in which we found the apolar patches to be conserved, while the anchors drastically switch their physico-chemical nature and drive major specificity changes, as illustrated in Figure 5. Further mutations outside the anchoring sites can then shape more exquisitely the specificity profiles.

The evolutionary constraints we have unraveled bring crucial information on how to improve scoring potentials for predictive docking using evolutionary information. Protein-protein interface prediction methods have been developed by combining evolutionary conservation and structural similarity, such as PRISM, which relies on the architecture of a template protein complex [57]. Our analysis provides very complementary insights since it aims at evaluating the likelihood of a protein-protein interface model by integrating as much evolutionary information as we found that was reliable, but without the use of a template interface. The predictors that we developed can be directly used to weight the contribution of various positions of a multiple sequence alignment to assess the likelihood of a structural model over the course of evolution. We propose that a hierarchy of rules should be applied when analyzing a structural interface model using the information brought by multiple sequence alignments. First, strong constraints should be applied to ensure that positions corresponding to anchors and apolar patches maintain a high complementarity in every homologous sequence. Outside these positions and given the prevalence of positions likely to switch out of interfaces, most concern should be directed toward core and support regions. Polar contacts were found to evolve in a versatile manner; yet, our quantification of the possible recovery scenarios can be used to rate the compatibility between a predicted interface and its evolutionary history. Altogether, these guidelines will provide highly valuable clues to better exploit the wealth of information contained in multiple sequence alignments towards prediction, as is the case for protein monomeric folds.

## Methods

### Detection of interface residues and sub-regions

Several procedures for the calculation of a protein-protein interface have been used in previous studies. The most popular definition relies on the variation in solvent-accessible surface area (rASA) upon binding [58]–[60], but there are alternative definitions based on residue environment [61] or interface contacts [11]. Following the latter definitions, interface residues were detected as those which either gain at least one structural neighbor upon complexation or make at least one contact with a residue in the other chain. Two residues are considered structural neighbors if their Cβ atoms (Cα for glycine) are within 8 Å of one another.

Three interface sub-regions were defined depending on the number of neighbors a residue had and the number of neighbors it gained upon binding [11], [39]. Two residues are defined as extended structural neighbors if their Cβ atoms are within 10 Å of one another; for glycine residues, we consider Cα atoms instead of Cβ. For each residue, the numbers of extended structural neighbors in the monomer and in the complex are calculated, and a burial index is then calculated for both the monomer and the complex as 0 if the residue has 15 neighbors or less, 1 if the residue has 24 neighbors or more, and a fraction in between (1/9 for 16 neighbors, 2/9 for 17 neighbors and so on). Then, if the difference in burial index between the monomer is over 0.5, the residue is considered “core” (it becomes significantly more buried upon complexation); else, if the average of the monomer and complex burial indices is over 0.6, the residue is considered “support” (significantly buried in both the monomer and the complex), otherwise it is considered “rim” (not very buried even in the complex). Details are available in Figure S3 in Text S2.

### Calculation of interface contact conservation

For each pair of interfacial residues in contact in one interolog, the corresponding pair of residues in the other interolog was identified thanks to the structural alignment (performed using MATRAS [62]) and we assessed whether this other pair of (structurally equivalent) residues was also in contact (Figure 1A). Only those residues with a structural equivalent in the interolog (determined on the basis of the structural alignment) were retained in the definition of interface contacts and their properties.

For each type of contact, the conservation is calculated as the ratio of the number of conserved contacts over the sum of the numbers of conserved and non-conserved contacts. This corresponds to the Jaccard index (similarity coefficient) between the graphs of contacts in each interolog. For atomic contacts and apolar contacts, if a contact between two positions exists in both interologs, the corresponding edge of the graph is weighted by the average number of atomic contacts between the two positions; if the contact exists in only one of the two interologs, the edge is weighted by the number of atomic contacts in the interolog where the contact exists.

In order to avoid detecting non-conservation effects due to gaps in the structural alignment of a pair of interologs, interfaces were restricted to residues which were structurally aligned with another residue in the interolog. We measured the sequence divergence of each pair of interologs using the minimum sequence identity at interface [9].

### Calculation of interface contacts, apolar contacts and apolar patches

Atomic contacts were calculated on the basis of an α-shape representation of the interface [63]. To compare contact conservation between two interologs, atomic contacts were grouped depending on the residues they involved. Charged contacts were defined as contacts between a N atom from arginine, lysine or histidine and an O atom from aspartate or glutamate within a 6 Å distance, salt bridges as charged contacts with a distance threshold restricted to 3.5 Å. Hydrogen bonds were defined by HBexplore with standard criteria [64]. Apolar contacts were calculated using the α-shape representation of atomic contacts, including all C and S surface interface atoms belonging to interface residues and using a van der Waals radius expansion of 0.7 Å (half the standard probe size) for polar atoms [43], [44]. Apolar patches for each side of the interface were defined as contiguous regions (based on the α-shape connections between surface interface C and S atoms and using a van der Waals radius expansion of 1.4 Å i.e. one standard probe size for polar atoms) containing at least four atoms from at least two different residues. To enable comparison between interologs, patches were iteratively merged so that one patch corresponded to a maximum of one patch in the interolog. Two patches (one on each side of the interface) were considered in contact if there was at least one apolar contact between them. Details are given in Text S1 (sections 8 and 18) and Figure S7 in Text S2.

### Definition of anchor residues

The notion of anchor provides a simple way to identify important residues for the interaction using geometric criteria [46]. In the present study, for each interface, we picked anchor residues as up to three residues from the core and support regions which bury the most solvent-accessible surface area (rASA) upon binding and in any case, more than 80 Å^2^. rASA values were assessed for each residue using NACCESS [65]. Details are given in section 11 of Text S1 and Figure S8 in Text S2.

### Evolutionary rate of interface residues

For each pair of structural interologs, a pair of multiple sequence alignments was generated using InterEvolAlign [36]. The evolutionary rates of interface residues were computed using the Rate4Site algorithm [3]. The conservation scores were normalized between 0 and 100 for each chain. The analysis was restricted to evolutionary rates calculated from multiple sequence alignments containing more than 10 sequences. Details are given in section 19 of Text S1.

### Statistical analysis, logistic regression and graphics

The R package was used to perform statistical tests, as well as to build and analyze the logistic regression models and assess the importance of the various parameters [66]. All the p-values presented in this paper were calculated using non-parametric Wilcoxon rank sum tests.

The confidence intervals in Figure 2 and Figures S7D and S8F in Text S2 were obtained by performing a bootstrap on the population of 1,024 interolog couples. This consisted in randomly drawing one half of the dataset (without replacement) one thousand times, calculating the mean value of contact conservation in each of the 1,000 resampled populations and extracting the intervals containing 95% of the calculated mean values.

Two predictors, one for the switching out of the interface, and another one for the conservation of atomic contacts, were built on the basis of the various interface features described in the Results section. Both predictors relied on a simple logistic regression to fit the coefficients corresponding to the chosen parameters and their quality was assessed on the basis of a ROC curve. The interface residues were split into a training dataset (interface residues from one third of the interolog couples drawn at random) and a test dataset (interface residues from the remaining two thirds of the interolog couples). This random partition of interfaces was repeated ten times. Each time, the logistic regression was performed on the training dataset and each residue in the test dataset was scored on the basis of the coefficients obtained in the regression. The residues in the test dataset were then ordered from the best score to the worst score and a ROC curve was drawn by progressively including all residues from all interolog couples, starting from residues with the best score towards residues with the worst score. The relative importance of all 6 parameters in both predictors as well as their significance were assessed on the basis of standard tests of deviance (see section 14 in Text S1 and Table S2 in Text S2).

## Supporting Information

Dataset S1 Dataset describing all 1,024 pairs of interologs used in this study, as well as all 387 pairs of complexes in the *redundant95* dataset. This dataset contains information such as interface sequence identity, structure resolution, interface RMSD, as well as the non-obligate or obligate nature of the interfaces, the likely paralogs and orthologs among interolog pairs, and the complexes contained in the subset of 60 transient interolog pairs.
(XLS)

Text S1 Supplementary results (sections 1 to 14), materials and methods (sections 15 to 20).
(PDF)

Text S2 Supplementary tables (4 tables) and figures (8 figures).
(PDF)
