# Supplementary material for: Versatility and Invariance in the Evolution of Homologous Heteromeric Interfaces
Source: PLoS Comput Biol. 2012 Aug 30;8(8):e1002677. doi: 10.1371/journal.pcbi.1002677 (PMC3431345; doi:10.1371/journal.pcbi.1002677)
Supplement: Text S1 — Supplementary results (sections 1 to 14), materials and methods (sections 15 to 20). (PDF) [file pcbi.1002677.s002.pdf]

Text S1 – Supplementary results, materials and methods

A. Supplementary results 2
1. Properties of the interfaces contained in the dataset of interologs 2
2. Global atomic contact conservation. 2
3. Switching out of the interface 3
4. Conservation of interface salt bridges. 5
5. Conservation of interface hydrogen bonds 5
6. Satisfaction of potential hydrogen bonds 6
7. Connectivity of salt bridges and hydrogen bonds 7
8. Properties of apolar patches 8
9. Conservation of interface apolar contacts 8
10. Conservation of apolar patches and their contacts, random reference for apolar patches 9
11. Conservation of anchor residues and their contacts 10
12. Overlap between anchor residues and apolar patches 12
13. Structural flexibility vs. evolutionary flexibility 12
14. Predictors of the switching out property and the atomic contact conservation 13
B. Supplementary materials and methods 16
15. Datasets 16
16. Definition of atomic contacts 18
17. Notion of geometric center of the interface, of an apolar patch 19
18. Definition of apolar contacts and apolar patches 19
19. Multiple sequence alignments and evolutionary rate of interface residues 20
20. Statistical analysis, logistic regression and graphics 21
References 22

A. Supplementary results

1. Properties of the interfaces contained in the dataset of interologs

Supporting Table 1 in Text S2 provides a detailed analysis of interface composition based on both the number of residues and the contribution to the interface area, for each sub-region and for the different categories of interfaces. These can be compared to the results drawn from [1]. The detailed figures are slightly different, probably because of the size and composition of the datasets; however, the overall tendencies are in good agreement. For instance, charged residues tend to be more frequently located in the rim than in the core of the interface. The composition of each interface on average includes 20 core residues, 14 support residues and 29 rim residues (respectively 16, 11 and 24 for non-obligate interfaces). Each interface in our database includes an average of 11 hydrogen bonds (including backbone-backbone H bonds), which represents on average 1 hydrogen bond per 80 $Å^2$ of polar interface area. Again, these numbers are consistent with the information provided for heteromeric complexes in [1,2].

2. Global atomic contact conservation.

In the whole interolog dataset, each interface has between 10 and 1597 atomic contacts (230 on average with a standard deviation of 181), corresponding to a range of 3 to 452 residue-residue contacts (61 on average with a standard deviation of 49). Non-obligate interfaces (as predicted by the NOXclass algorithm [3]) have a smaller number of atomic contacts than obligate interfaces.

Strong contacts between residues which involve several atomic contacts should be more difficult to lose between interologs than weaker contacts. This is why we considered atomic contacts grouped by residue, instead of contacts between residues on a simple pairwise basis. With residue-based contacts, the average conservation is 52.3% instead of 59.3% with weighted contacts (p-value < 2.2e-16).

The contact conservation analysis does not take into account the type of residues in contact: the contact can be conserved even if the amino acids are different in the two interologs, as the correspondence between residues in the interolog pair is based on structural alignment only. On the other hand, a contact can be lost even if the amino acids do not change, because the side chains can be placed differently in the two interologs. There are around 15% of non-conserved contacts in *redundant95* but by definition, very few (only 5.5% of the non-conserved contacts) come from mutations, so at least part of the lost contacts do not result from the difference in amino acid composition. In the whole interolog dataset, 12% of the non-conserved contacts involve no amino acid mutation. This reflects the fact that the notion of conservation is not too strict, because it is independent from the exact amino acids involved in the contact.

We observed that there was no variation in the contact conservation with respect to secondary structure. Each residue in the datasets was assigned a secondary structure using DSSP [4] which provides a uniform definition of secondary structure elements which allows for subsequent characterization and comparison of interologous interfaces. Focusing on contacts involving either two structurally aligned residues which both have a predicted secondary structure (making up 57% of all contacts) or two structurally aligned residues with no

predicted secondary structure (making up 27% of all contacts), as well as contacts including residues involved in the same type of secondary structure (α-helices or β-ladders and sheets), no significant difference was observed (p-value > 0.09), except for the conservation in β-ladders and sheets which seems slightly higher than average (p-value = 0.01) and slightly higher than the conservation in α-helices (p-value = 0.01).

Is there a difference in the conservation of atomic contacts between non-obligate and obligate complexes? When analyzing the two separately (as predicted by NOXclass [3]), there is no statistically significant difference between the distributions of conservation among interolog pairs (p-value = 0.84). The distributions of non-obligate and obligate conservation as a function of sequence identity at interface are plotted in Supporting Figure 4A in Text S2. The non-obligate distribution is significantly different from the obligate distribution only for interface sequence identities 30-70% and even then, the significance level is quite low (p-value = 0.03 for 30-50% and 0.02 for 50-70%).

We checked that this absence of significant difference was not a simple consequence of the method used to predict obligate and non-obligate interactions, by studying the influence of the manually assigned non-obligate or obligate character of the interface (see supplementary methods on datasets). When the distributions of atomic contact conservation for the manually curated non-obligate and obligate datasets are compared, the results are very similar to the comparison between the non-obligate and obligate complexes as predicted by NOXclass [3].

Besides, we wondered whether there was a difference in contact conservation between orthologous and paralogous pairs of interologs. After manual curation, two datasets were obtained containing likely orthologs and likely paralogs (see supplementary methods on datasets), for which atomic contact conservation was compared. Contacts are more conserved on average for orthologs than for paralogs: the average contact conservation is 62.9% for orthologs and 57.1% for paralogs. The distributions are significantly different (p-value = 1.6e-3). However, there is a strong dissymmetry in the distribution of orthologs and paralogs with respect to sequence divergence: there are many more paralogs than orthologs at low sequence identities because the complexes have diverged in function. Supporting Figure 4B in Text S2 thus displays the distributions of contact conservation depending on sequence identity. The distributions for orthologs and paralogs are not significantly different (p-value = 0.67 for 0-30%, 0.33 for 30-50%, 0.44 for 50-70% and 0.99 for 70-100%).

3. Switching out of the interface.

We checked that the "switching out" was not a consequence of the choice to detect interface residues on the basis of inter-molecular contacts and residue environment by redefining the interface on the basis of solvent-accessible surface area criteria. For this verification purpose, the core, support and rim regions were also redefined using the definitions proposed in [5]. The distribution of the proportions of residues switching out of the interface for the whole interolog datasets as well as the distributions corresponding to the contributions of the core, support and rim regions are not significantly different between the two interface definitions (p-value > 0.48 in Wilcoxon rank sum tests).

When considering residue-based contacts, "switching out" of the interface accounts for 37.5% of the non-conserved contacts for the whole dataset and 17.6% of the non-conserved contacts in *redundant95*. The fact that the proportion of residue-based contacts lost due to switching

out is close to the proportion of atomic contacts lost due to switching out shows that the positions switching out of the interface are not only residues making “weak” contacts with very few atoms involved. However, in the switching out predictor (see main text and section 14 below), we see that the number of atomic contacts is an important parameter to determine whether a residue is likely to switch out of the interface in the interolog.

The distributions of the distance to the atomic center (see supplementary methods) for the different sub-regions of the interface show that the rim residues are both the most involved in the “switching out” and the furthest from the atomic center. As illustrated in Supporting Figure 3A in Text S2, the central residue (the residue closest to the geometric center) of each side of the interface remains at a structurally equivalent position in 56% of interologs and moves to a neighboring position in 30% of all interologs. The modifications occur mostly in the contours of the interface, with no “sliding” of the interface. Thus, the definition of interface sub-regions is a good indicator of the probability that a residue will switch out of the interface, and the distance to the geometric center is a simple descriptor expressing the same trend. In the switching out predictor (see Main Text and section 14 below), the sub-region of the interface (core, rim, support) is found to be the most important descriptor and the normalized distance to the geometric center of the interface is found to be a complementary descriptor with a smaller but significant effect.

How to interpret switching out occurring in the core? First of all, core residues switching out of the interface are quite rare (7% of all core residues compared to 30% of support residues and 33% of rim residues). Four major factors were found to account for their likelihood to switch out. Overall, core residues switching out of the interface are further away from the geometric center of the interface than core residues remaining in the interface (means of normalized distance to the geometric center: 0.54 vs 0.46, p-value < 2.2e-16). They also correspond to cases of higher flexibility (49% of the core residues switching out of the interface have no involvement in a secondary structure element or are involved in a bend). Third, high iRMSD between 2 interologous structures tends to induce switching out of core residues (44% of the core residues switching out of the interface correspond to a pair of interologs with over 4Å iRMSD). Last, 8% of core residues switching out actually have a very low accessibility difference (<= 5 Å2, low involvement in the interface). If we combine these 4 criteria (distance to the geometric center of the interface, secondary structure, iRMSD, accessibility difference) we are left with only 5% of switching out core residues that correspond to other explanations.

Which residues/contacts switch out more? Charged residues are more likely to switch out of the interface, in particular acidic residues and lysine (arginine to a lesser extent). This is consistent with the types of contacts that switch out more (switching out accounts for 36% of the non-conservation for salt bridges, 27% for hydrogen bonds, 25% for apolar contacts). More generally, the switching out is a combination of two effects: a rim effect (charged/polar residues at the periphery of the interface) and a support effect (rather apolar residues in the support region which become even more buried in the interolog, to the point that they are no longer surface-accessible).

How often is switching out connected with a sequence change? On average, for 51% of all interface residues, the aligned residue in the interolog is the same amino acid. 64% of switching out residues are connected with a sequence change. However, about half of the non-switching out residues (46 %) are also connected to sequence changes. Thus, there is a differential effect of sequence change, but this effect is of limited amplitude and cannot

explain alone all switching out situations. This property was included in the switching out predictor and exhibited a significant contribution (see below, section 14).

4. Conservation of interface salt bridges.

In the whole interolog dataset, a given interface has between 0 and 14 salt bridges with an average of 2 salt bridges per interface. 15% of all interolog pairs contain no salt bridge in either of the two interfaces and 23% of all interolog pairs contain at least one salt bridge in one of the two interfaces and no salt bridge in the other interolog (in this case, the interolog with salt bridges has on average 1.7 salt bridge). Finally, 21% of all interolog pairs contain the same number of salt bridges (excluding the pairs that both have no salt bridge).

In addition, salt bridges occur between the exact same amino acids in over 82% of the conservation cases in the interologs dataset: switches between lysine and arginine residues are quite rare and the same is true between aspartate and glutamate.

There is no obvious correlation between the conservation of salt bridges and secondary structure, or between the conservation of salt bridges and the non-obligate or obligate nature of the interaction, either with the NOXclass definition [3] or the manually assigned data (even when we split into four sequence divergence categories, p-value > 0.08). As before, when distinguishing between orthologs and paralogs, if the data is split into four interface sequence identity ranges, there is no significant difference in salt bridge conservation between orthologs and paralogs (p-value > 0.11 for 0-30%, 30-50%, 50-70% and 70-100%).

Supporting Figure 3E in Text S2 (red distributions) shows that the core and support regions have higher salt bridge conservation than average and the rim region lower salt bridge conservation. The rim distribution is statistically different from the core and support distributions (p-value < 7.3e-5).

5. Conservation of interface hydrogen bonds.

Almost all interfaces contain at least one hydrogen bond. On average, there are 11 hydrogen bonds per interface. About 19% of all hydrogen bonds in all interologs occur between backbone atoms, whereas 36% occur between side chain atoms and 45% between one side chain atom and one backbone atom.

When H bond conservation was analyzed without distinguishing between the various types of H bonds, on average, 35.7% of H bonds were found to be conserved between interologs. However, this reflects a mixture of very different conservation patterns: H bonds between backbone atoms are much more conserved than H bonds involving one or two side chain atoms, but they represent a minority and do not reflect the same interface plasticity as H bonds involving side chains. As a consequence, in the conservation analysis, only H bonds involving at least one side chain atom were considered. This leaves an average of 9 hydrogen bonds per interface included in the conservation calculation. This leads to a lower, but more accurate “restrictive conservation” of 27.8%. This “restrictive conservation” is the basic measure of conservation used throughout the main text of the paper and the rest of the SI.

Supporting Figure 3E in Text S2 (blue distributions) shows that the core and support regions have higher H bond conservation than average and the rim region lower H bond conservation. The rim distribution is significantly different from the other two (p-value < 2.2e-16) and the support distribution is significantly more conserved than the core distribution (p-value = 2.6e-3).

Hydrogen bonds between residues with a normalized Rate4Site conservation score over 80 are more conserved than other hydrogen bonds: the conservation increases from 27.8% to 47.1% on average.

What happens in the cases of non-conserved hydrogen bonds? (Supporting Figure 6C-D in Text S2) Non-conserved hydrogen bonds correspond to at least one residue switching out of the interface in 27% of all non-conservation cases. In 40% of the non-conservation cases, at least one residue whose side chain was involved in the H bond undergoes a mutation which makes the new side chain unable to participate in a hydrogen bond. In 33% of cases, both residues remain polar and at interface. Moreover, when a residue belonging to a non-conserved hydrogen bond remains polar and at interface, in a vast majority of cases (78%), it is involved in at least one other intra- or inter-molecular H bond.

There is no obvious effect on hydrogen bond conservation of secondary structure, the non-obligate or obligate nature of interfaces, or the orthologous or paralogous relationship between interologs.

6. Satisfaction of potential hydrogen bonds

In the monomeric core, residues with polar atoms prone to hydrogen bonding almost always make H bonds [6]. As hydrogen bonds were poorly conserved between interologs, we wondered to what extent residues with polar atoms satisfied their hydrogen bonding potential in the interfaces of the dataset. We found that for interfaces, the proportions of unsatisfied donor and acceptor atoms are higher than for atoms buried in the monomeric core; however, detailed examination of the unsatisfied atoms in several examples revealed that in most cases, the atoms are hydrogen bonding to interface water molecules (details below). There remain few unsatisfied polar atoms at interface, which confirms that the hydrogen bonding constraint is quite strong.

For this study, we focused on individual structures with a resolution better than 2.0 Å (318 interfaces in the whole dataset). It was checked that inside the monomeric chains of the interologs, hydrogen bond donor and acceptor atoms that are buried (in the monomer) satisfy a large majority of their H bonds, and these results were compared to [6]. 13.0% of buried backbone donor atoms, 7.6% of backbone acceptor atoms, 12.1% of side chain donor atoms and 26.0% of side chain acceptor atoms fail to hydrogen bond. These values are higher than those found in [6] and this difference can be explained by the fact that hydrogen bonds to the solvent were not considered here. However, similar tendencies to those found in [6] were uncovered in the behavior of each residue type and the frequent non-saturation of multiple hydrogen bonds.

All hydrogen bond donor and acceptor atoms were then extracted from interface residues with an accessibility difference between the free and the bound forms (“interface atoms”). We focused on N and O atoms because S atoms have been shown to have a low level of hydrogen bonding [6]. Mainly atoms exposed in the unbound form of the complex but buried in the

complex (accessibility = 0) were considered, because atoms that are already buried in the monomeric form of each chain probably satisfy their hydrogen bonds intra-molecularly and atoms that are solvent-exposed in the complex can make hydrogen bonds with the solvent.

Checking how many of the potential hydrogen bonds were actually satisfied, we found that the proportion of unsatisfied donor and acceptor atoms was higher than for atoms buried in the monomeric core: 18.6% of buried backbone donor atoms, 17.6% of backbone acceptor atoms, 11.0% of side chain donor atoms and 26.1% of side chain acceptor atoms fail to hydrogen bond. Detailed examination of the unsatisfied atoms in several examples revealed that in more than half of the cases, the atoms are hydrogen bonding to interface water. There remain some unsatisfied polar atoms at interface, but in very small proportions, which confirms that the need for hydrogen bonding is quite strong and provides an energetic interpretation of the fact that if the residues involved in a H bond lost in the interolog remain polar, they will in most cases hydrogen bond to other residues.

7. Connectivity of salt bridges and hydrogen bonds

The connectivity of polar contacts was assessed in order to see if networks of polar contacts could be defined, which could play an important role in explaining the versatility of these contacts. The aim was to find a way to describe polar contacts which would be analogous to apolar patches with respect to apolar contacts.

Networks of interface (inter-chain) hydrogen bonds were built. For each interface, a graph was built where the edges were all inter-chain H bonds and all (except backbone/backbone) intra-chain H bonds. The connected components (networks of residues connected by H bonds) were extracted from this graph and the way such networks were transposed from one interolog to the other was assessed. To this effect, inter-molecular backbone/backbone bonds were re-integrated so that they could link two residues that might otherwise be artificially disconnected in the network. Intra-molecular H bonds were also integrated (except for backbone/backbone bonds which are not specific and too abundant). H bond networks were characterized. 55% of them contain only two residues, 20% contain 3 residues, 10% contain 4 residues, 5% contain 5 residues and the remaining 10% of networks contain 6 residues or more. For each pair of interologs, a residue which is in a network in one of the interologs has 62% of chances of being in a network in the other interolog as well. However, when the analysis was restricted to residues belonging to a network of a certain size, the larger the network, the higher the probability that the residue would be involved in a network (whatever its size) in the interolog: for instance, if a residue belongs to a network of 3 residues or more (respectively 4 residues or more), it has 67% (respectively 70%) of chances of being in a network in the interolog.

Thus, trying to build networks of interface hydrogen bonds, we found that half of them contained only two residues. Therefore, the notion of network itself is not very helpful to describe the fate of non-conserved polar contacts. We thus wondered, when a residue is involved in a polar bond in one interolog and in a different polar bond in the other interolog, how far we needed to reach in order to recover the bond. In other words, what is the sphere of influence which a residue can span in order to recover a lost polar bond? Similar results were obtained for hydrogen bonds and salt bridges: on average, in 57-60% of cases, the two bonds involve residues that are immediate neighbors (in the sense of a sphere of neighbors with a 7 Å radius which includes on average 6 or 7 residues) and in 29-30% of cases the two bonds

involve residues that are located within the second sphere of neighbors of each other (in the sense of a sphere with 12 Å radius which includes more than 20 residues on average). The predictive power in the case of polar bond recovery is thus limited.

8. Properties of apolar patches

Patches of apolar atoms are clustered through the edges of the apolar contact network. Clustering is controlled by the spatial distribution of polar atoms which tend to break the apolar interconnections. The construction of apolar patches is explained in detail below in section 18 of the supplementary methods below.

Apolar patches were defined using a minimum threshold of four atoms coming from at least two different residues. The motivation in establishing this threshold was to be more specific while retaining as much information as possible. If no threshold is set, 89% of all interface residues are involved in a patch through at least one of their atoms and only 70% of apolar patches have an equivalent patch in the interolog because many patches are very small and quasi-artifacts due to the broad definition of apolarity. The different possibilities were carefully examined and it was decided to set a threshold in the number of residues involved in the patch. The 4-atom and 2-residue threshold was a good compromise between the specificity of apolar patches (with this threshold definition, 41% of all interface residues are involved in a patch through at least one of their atoms), the representativeness of apolar patches (only 5% of all chains in all complexes have solely apolar patches containing strictly less than 2 residues and 4 atoms, so that they have no apolar patch with this threshold definition) and the correspondence of patches between interologs (with this threshold definition, 82% of patches have an equivalent in the interolog).

On average, in the dataset of interologs, an interface contains between 2 and 5 apolar patches on each side of the interface, each patch contains an average of 3.8 residues and 41% of all interface residues are involved in an apolar patch through at least one of their atoms. 99% of apolar patches are actually involved in at least one apolar contact at interface.

Because patches were defined to be equivalent between two interologs if they involved at least one common position (structurally aligned between the two interologs), it was checked that the patches termed equivalent had a similar composition in terms of the positions involved and corresponded to similar contours. If two equivalent patches in a pair of interologs are considered, there are limited fluctuations in the contours of the patch: only 7.6% of residues belonging to a patch which has an equivalent in the interolog “switch out” of the patch in the interolog. In patches with an equivalent in the interolog, residues that “switch out” of the patch are further away from the geometric center of the patch (see supplementary methods) relative to residues which stay in the patch in both interologs (p-value = 2e-6).

9. Conservation of interface apolar contacts

To start with, a simple definition of apolar contacts relying on hydrophobic amino acids only was tested. Interfaces contain an average of 10 apolar residue contacts of this type, each involving on average 3.2 atomic contacts and on average, 39.4% of apolar contacts are conserved between interologs if we use the definition based on hydrophobic amino acids only. However, using a more elaborate description of apolar contacts relying on apolar atoms belonging to any residue type implied that interfaces contained more apolar contacts (on

average, 35 residue contacts each involving 2.5 atomic contacts) and contact conservation was higher (51.3% on average). This illustrates the interest of using a broad definition of apolarity in order to assess contact conservation. All the results described in the main body of the paper are calculated using this description of apolar contacts relying on all apolar atoms from any type of amino acid.

Supporting Figure 3E in Text S2 shows the distributions of conservation for contacts involving structurally aligned residues from the same sub-region of the interface. For each type of contact, including apolar contacts (in golden yellow), the conservation is higher in the core and support regions and lower in the rim region. However, whatever the sub-region of the interface, and in particular in the rim region, apolar contacts are more conserved than polar contacts. The core and support regions have higher apolar contact conservation than average and the rim region lower contact conservation. The differences between the rim distribution and the support and core distributions are statistically significant (p-value < 2.2e-16) but the difference between contacts involving residues from the support and the core regions is not significant (p-value = 0.64).

Secondary structure has no significant influence on the conservation of apolar contacts. There is not either any obvious difference in apolar contact conservation between non-obligate and obligate interfaces using the NOXclass prediction (p-value = 0.39). There is no significant different in apolar contact conservation for manually curated non-obligate and obligate interfaces (p-value = 0.94). Finally, between orthologs and paralogs, overall, apolar contacts are more conserved in orthologs (54.7% of conserved apolar contacts on average) than in paralogs (49.4%) (p-value = 2.3e-3), but if the data is split into four ranges of interface sequence identities, it appears that the difference comes mainly from the fact that paralogs are more divergent than orthologs.

However, 67.6% of pairwise apolar contacts between residues with a Rate4Site normalized conservation score above 80 are conserved on average, which represents a significant increase for all ranges of interface sequence identity (p-value < 4.8e-3). This highlights again the interest of using slowly evolving positions as a marker of contact conservation.

10. Conservation of apolar patches and their contacts, random reference for apolar patches

49% of apolar contacts occur between residues involved in apolar patches, 40% between one residue involved in an apolar patch and one residue not involved in a patch, and 11% between two residues not involved in apolar patches. As illustrated in main Figure 2, apolar contacts between two residues both involved in apolar patches are significantly more conserved than apolar contacts between one residue in a patch and one residue not in a patch, which are in turn significantly more conserved than apolar contacts between two residues not involved in patches.

We defined a random reference for apolar patches, to assess their significance and specificity. We created random patches with a distribution of patch size and distributions of core, support and rim patch residues as close as possible to the distributions for the real apolar patches. The

detailed procedure was the following: for each real apolar patch, we created a corresponding random patch. To this effect, we defined the central residue of the real patch as the residue closest to the geometric center of the patch and we assessed to which sub-category of the interface this residue belonged (core, support or rim). Then we picked a residue from this sub-category at random as the central residue of the corresponding random patch. We expanded the random patch around this residue: until the random patch had the same number of residues than the real patch, we randomly picked residues in the first shell of neighbors around the central residue; if all residues from the first shell had been picked and the size of the random patch had not reached the size of the real patch, we proceeded to picking more residues from the second shell of neighbors; then again if necessary, from the third shell, and so on. If the corresponding real apolar patch had no equivalent apolar patch in the interolog, then no equivalent random patch was defined. If the corresponding real apolar patch had an equivalent apolar patch in the interolog, then an equivalent patch was also defined for the random patch: following what happens for real apolar patches, in 80% of cases the residue structurally equivalent to the central residue of the patch in the first interolog was picked as central residue of the patch in the second interolog, in 20% of cases a direct neighbor was picked. The patch was then expanded in the second interolog around this central residue, just as in the first interolog.

Please note that we never constrained the random patches not to overlap with the real apolar patches, so that it can very well happen that a random patch has some residues in common with a real apolar patch. Because of the way the random patches are built, they follow the same size distribution and contain the same proportions of core, support and rim residues as the real apolar patches. However, the residue composition is much more biased towards hydrophobic residues for real apolar patches than for random patches. Random patches contain 41% of hydrophobic residues (MVLIFWYP) whereas real apolar patches contain 71% of hydrophobic residues.

We verified that the conservation of apolar contacts between random patches is significantly lower than the conservation of apolar contacts between real apolar patches: on average, the percentage of conserved apolar contacts between patches is 58% for random patches vs. 84% for real apolar patches (p-value between the distributions of contact conservation in a Wilcoxon rank sum test $< 2.2e-16$). This further underscores the specificity of apolar patches.

Moreover, we controlled that random patches faced each other in a manner similar to that occurring between naturally observed apolar patches. Real apolar patches which are in contact are connected through an average of 3.8 residue-residue contacts (defined by grouping atomic contacts into residue-residue contacts). Random patches as defined above are connected through an average of 3.4 residue-residue contacts. We checked the conservation of contacts between random patches if we picked a subset of the random patches in which the patches in contact had the same average number of residue-residue contacts (3.8) than the real apolar patches – this was achieved by eliminating pairs of patches that were connected through one or two residue-residue contacts. With this additional constraint, the conservation of apolar contacts between random patches was increased (from 58% to 63%) but the conservation of apolar contacts between real apolar patches remained significantly higher (84% vs. 63%, p-value $< 2.2e-16$).

11. Conservation of anchor residues and their contacts

Supporting Figure 8D in Text S2 shows the distribution of variation in solvent-accessible surface area for the 20 residues with the highest variation, ordered by decreasing variation, starting from the residue with highest variation. The residue with highest variation has a distribution that is visibly higher than others; however, for some interfaces where the residue with highest variation has variation under 100 $Å^2$, the second and third residue with highest variation might also be important for the interaction [7,8].

For the sake of clarity, a single definition of anchor residues was chosen which is the following: for each interface, we take up to three residues from the core and support regions which have the highest difference in solvent accessibility between the free and the bound forms and in any case, a difference in solvent accessibility higher than 80 $Å^2$. This means that the anchor residues are necessarily from the core and support region (99% core, 1% support) and bury the largest amount of solvent-accessible surface area in the interface (and in any case, more than 80 $Å^2$).

There are 9% of all interfaces in the interolog dataset for which there are no such residues, 9% of all interfaces for which only one anchor residue can be defined, 11% of all interfaces for which two anchor residues can be defined. The remaining 71% of interfaces have 3 anchors.

Anchor residues make more interface contacts than other core residues: on average an anchor residue makes 24 atomic contacts (with a standard deviation of 10) and other core residues make 10 atomic contacts (with a standard deviation of 7). The distributions of the number of atomic contacts are significantly different between anchor residues and other core residues (p-value < 2.2e-16).

Results for all anchor residues

For a fair comparison of anchor residues with core and support residues, we compare the conservation of contacts involving at least one anchor residue (in any of the two interologs) with the conservation of contacts involving at least one core or support residue (in any of the two interologs). This comparison is represented in Supporting Figure 8E and 8F in Text S2.

When considering only contacts involving anchor residues, the average atomic contact conservation is raised from 63.2% for core and support residues to 67.7% for anchor residues and this increase is statistically significant (p-value = 3.7e-8). The average salt bridge conservation is not significantly higher in anchor residues compared to core and support residues. However, there are very few salt bridges per interface and when the analysis was limited to anchor residues, an even smaller population of bonds was studied which might be difficult to statistically compare to the original population. Moreover, salt bridges more often involve rim residues than other types of contacts. The average H bond conservation is raised from 30.1% for core and support residues to 38.1% for anchor residues and this increase is significant (p-value = 7e-4). Finally, the average apolar contact conservation is increased from 55.3% for core and support residues to 59% for anchor residues and this increase is statistically significant (p-value < 4e-5).

Results for anchor residues whose structural equivalent is also an anchor residue

53% of anchor residues conserve their anchor character in the interolog.

The conservation of contacts involving at least one anchor residue whose structural equivalent in the interolog is also an anchor residue (whatever the nature of the residues) was studied. The average atomic contact conservation is raised from 67.7% (for all anchor residues) to 82.9% and this increase is statistically significant (p-value <2.2e-16). Salt bridge conservation is 40% for anchor residues whose structural equivalent is also an anchor residue, but such residues participate in very few salt bridges. The average hydrogen bond conservation is further increased to 46.6% and this increase is statistically significant compared to all anchor residues (p-value = 3e-3). Finally, the average apolar contact conservation is increased to 72.4% and this change is statistically significant compared to all anchor residues (p-value < 2.2e-16).

Detection of anchor residues whose structural equivalent is also an anchor residue

The amount of conservation for interface atomic and apolar contacts becomes high when the analysis is restricted to structurally equivalent anchor residues, and the conservation of polar contacts is much higher than average. However, anchor residues which are conserved as anchor residues in the interolog are hard to discriminate from non-conserved anchor residues based solely on standard criteria such as the type of residue.

Importance of anchor residues

Taking into account the properties of the core, the relative increase in anchor conservation is moderate but significant with respect to other core residues. The general trend is globally that the more contacts residues involve in an interface, the more their contacts tend to be conserved. This is consistent with the results we observed in developing the predictors (see below, section 14). Over the six features selected on their ability to improve the quality of the prediction, the number of contacts per residue was found to contribute significantly (as the third major factor for the contact predictor). Moreover, anchor residues were found to be significantly enriched in the residues predicted as most conserving their contacts, although none of the 6 features used explicitly included anchors or buried surface.

12. Overlap between anchor residues and apolar patches

48% of all anchor residues belong to apolar patches, compared to 41% of all interface residues. This slight enrichment is no higher than expected simply from the fact that anchors are core and support residues and apolar patches are enriched in core and support residues (core and support residues make up 70% of apolar patches vs. 58% of whole interface).

Among anchor residues involved in apolar patches, 51% conserve their anchor character in the interolog so we did not find more conservation of anchor residues within patches than outside patches.

13. Structural flexibility vs. evolutionary flexibility

From the compared analysis of the redundant dataset and the dataset of interologs, we extracted 52 cases where for a given complex, we have the structures of both a redundant complex (with over 95% sequence identity) and at least one structural interolog. Among these 52 cases, 44 groups of interologs are represented (1 with 3 cases with redundant complexes, 6 with 2 cases with redundant complexes and 37 with 1 case with a redundant complex). These 52 cases are listed in Supporting Table 3 in Text S2. Relying on these 52 cases, we compared the evolutionary flexibility (contact conservation between the central complex and its interolog(s)) and the structural flexibility (contact conservation between the central complex and its redundant complex) for each salt bridge and each apolar contact involved in the central complex. These represent 305 salt bridges and 5492 apolar contacts for which we assessed the conservation both in the interolog(s) and in the redundant complex.

Please note that due to the bias concerning complexes with several highly redundant structures available in the PDB, 16 interfaces represented in this set of 52 cases correspond to several interfaces between different subunits of large complexes (RNA polymerase II, F1-ATPase and cytochrome c).

For salt bridges, we find that when the salt bridge is still present in the redundant complex, then it is also present in the interolog in 35% of cases (lost in the interolog in 65% of cases); when the salt bridge is lost in the redundant complex, it is still present in the interolog in only 20% of cases (lost in the interolog in 80% of cases). For apolar contacts, we find that when the apolar contact is still present in the redundant complex, then it is also present in the interolog in 63% of cases (lost in the interolog in 37% of cases); when the apolar contact is lost in the redundant complex, it is still present in the interolog in 42% of cases (lost in the interolog in 58% of cases).

This means that the conservation of the contact in the redundant complex provides some information about the potential conservation of the contact in the interolog for both types of contacts. However the loss of the contact due to structural flexibility does not necessarily imply the loss of the contact through evolution. Moreover, the amount of information gained from the structural flexibility is not obviously different for salt bridges (35% compared to 20%) and for apolar contacts (63% compared to 42%).

14. Predictors of the switching out property and the atomic contact conservation

Two predictors were built on the basis of various interface descriptors, one for the switching out of the interface, and another one for the conservation of atomic contacts. The aim of the predictor is to rank, given an interface between 2 proteins A and B and sequence alignments of A and B with two homologous chains A' and B', which positions in the A'-B' complex are likely to switch out or conserve their contacts. Both predictors relied on a simple logistic regression to fit the coefficients corresponding to the chosen parameters and their quality was assessed on the basis of a ROC curve.

Various parameters were tested with respect to their influence on the quality of the predictors and 6 parameters were retained (the parameters are the same for both predictors and they are listed in main Table 1). None of the other factors, such as secondary structures of the residue

or its neighbors or Rate4Site evolutionary rates, were found to increase the predictive power significantly.

For both predictors, the dataset of all interface residues from the 1,024 interolog couples was randomly split into a training dataset containing the interface residues corresponding to one third of the interolog couples and a test dataset containing the interface residues corresponding to two thirds of the interolog couples. This random splitting procedure was repeated ten times. For the switching out predictor, ~132,200 interface residues were considered and split into a training dataset of ~44,000 residues on average (varying in the 10 repeats between 41,865 and 46,524 depending on the interolog couples picked) and a test dataset of ~88,200 residues on average (varying in the 10 repeats between 85,696 and 90,355 depending on the interolog couples picked). For the contact conservation predictor, only residues making at least one interface contact were considered, which resulted in considering ~115,000 interface residues, split into a training dataset of ~38,470 residues on average (varying in the 10 repeats between 34,840 and 42,256 depending on the interolog couples picked) and a test dataset of ~76,540 residues on average (varying in the 10 repeats between 72,759 and 80,175 depending on the interolog couples picked).

In each of the 10 repeats of the random partition into training and testing datasets, the logistic regression was performed on the residues in the training dataset (for which we know the result – either whether the residue switches out of the interface or not, or what proportion of its contacts it conserves between interologs) and each residue in the interfaces belonging to the test dataset was scored on the basis of the coefficients obtained in the regression (logistic regression coefficients, displayed in main Table 1). The residues in the test dataset were then ordered from the best score to the worst score and a ROC curve was drawn by progressively including all residues from all interfaces, starting from residues with the best score towards residues with the worst score.

Please note that for the switching out predictor, a high score means a high predicted probability to switch out of the interface, whereas in the contact conservation predictor, a high score means a high predicted probability for the residue to have the same contact network in the interolog (meaning that it is likely not to lose the contacts it participates in and not to gain other contacts).

The ROC curve represents the “fraction of true positives” vs. the “fraction of false positives” obtained in the course of the progressive inclusion of all residues. For the switching out predictor, “true positives” are residues which actually switch out of the interface and “false positives” are residues which do not switch out of the interface. For the contact conservation predictor, for each residue, “true positives” are the contacts involving this residue which are conserved (present in both interologs) and “false positives” are the contacts either lost by this residue (i.e. not present in the interolog anymore) or gained by this residue (i.e. contacts which are not present in the interolog to which the residue belongs, but which are present in the other interolog and in which the structurally aligned residue participates).

For the switching out predictor, the score for each residue is given by equation (E1). The area under the obtained ROC curve is 0.79 (for the ten repeats, values between 0.7915 and 0.7962 were obtained, with an average of 0.794 and a standard deviation of 0.001). The ROC curve for the switching out predictor is represented in Supporting Figure 5A in Text S2.

$$score_{switchingout} = -0.122 * nbcontacts + \begin{Bmatrix} 0 \text{ if } core \\ 0.96 \text{ if } support \\ 1.06 \text{ if } rim \end{Bmatrix} - 0.128 * similarity_{environment} + 1.40 * dist_{geomcenter} - 0.0096 * seqid_{global} - 0.054 * similarity_{residue} - 1.99 \quad \text{(E1)}$$

For the contact conservation predictor, the score for each residue is given by equation (E2). The area under the obtained ROC curve is 0.75 (for the ten repeats, values between 0.7447 and 0.7515 were obtained, with an average of 0.748 and a standard deviation of 0.002). The ROC curve for the contact conservation predictor is represented Supporting Figures 5B in Text S2.

$$score_{contactconservation} = 0.224 * similarity_{environment} + 0.075 * nbcontacts + 0.112 * similarity_{residue} + \begin{Bmatrix} 0 \text{ if } core \\ 0.23 \text{ if } support \\ -0.21 \text{ if } rim \end{Bmatrix} + 0.0066 * seqid_{global} - 0.69 * dist_{geomcenter} - 1.32 \quad \text{(E2)}$$

The importance of the parameters was estimated by dropping systematically one parameter at a time and estimating the deviance change (Likelihood Ratio Test) between the 6-parameter model and the remaining 5-parameter model. This was done using the drop1 function from the R statistics package [9]. These values are reported for both predictors in Supporting Table 2 in Text S2, together with their variability over the 10 repeats. For both predictors, all six parameters were found to be very significant both from the z-tests performed on the logistic regression coefficients and from the deviance test (with a chi-square null model): significance values were always found to be < 2.2e-16.

Finally, the parameters were ordered from the parameter contributing the most in the deviance test to the parameter contributing the least, and the deviance upon the progressive inclusion of one parameter after the other was analyzed. This was done using the anova function from the R statistics package [9]. The corresponding reductions in deviance are also reported for both predictors in Supporting Table 2 in Text S2 and they are in very good agreement with the results from the deviance test consisting in dropping one parameter at a time described above.

The large amount of data and the small number of parameters make it less likely that the model is overfitted. Moreover, we used a partition of the dataset into one third for training and two thirds for testing, which is quite challenging. However, to be more confident about the absence of overtraining in our logistic regression, we assessed the area under the ROC curve that would be obtained if the regression coefficients obtained from a given training dataset were used to evaluate the scores of residues from the same training dataset (instead of the test dataset). If this area value was very different from the value obtained with different training and test datasets, it would be a sign of overtraining.

For the switching out predictor, the corresponding area under the obtained ROC curve (for a prediction performed on the training dataset) is 0.79 (for the ten repeats, values between 0.786 and 0.800 were obtained, with an average of 0.792 and a standard deviation of 0.004).

For the contact conservation predictor, the corresponding area under the obtained ROC curve (for a prediction performed on the training dataset) is 0.75 (for the ten repeats, values between 0.737 and 0.754 were obtained, with an average of 0.746 and a standard deviation of 0.005).

Thus, the values obtained for the use of the training dataset as a test dataset and for the use of a test dataset strictly different from the training dataset are very close.

Importance of anchors in the contact conservation predictor

If we order residues on the basis of their score in the predictor of atomic contact conservation (i.e. the first residue is the one most likely to conserve its contacts), although none of the 6 features used explicitly included anchors or buried surface, we see a very significant enrichment of anchor residues among the best residues. Indeed, although anchors represent only 4.3% of the total number of residues, among the top 100 residues, there are 87 anchors (amounting to 1.7% of all anchors), and among the top 1000 residues, there are 532 anchors (amounting to 10.7% of all anchors vs. 0.9% of all residues). This is the sign that anchor residues display a very interesting combination of a "fundamentally core" character (the three residues burying the most solvent-accessible surface upon binding) and a very high number of atomic interface contacts.

B. Supplementary materials and methods

15. Datasets

The dataset of interologs is based on the structural interologs below 70% sequence identity extracted from the InterEvol database [10]. Details are given in [10] but here we give a summary of the relevant techniques. The dataset of interfaces extracted from the Protein Data Bank was filtered to remove redundancy and clusters of chains sharing more than 70% overall sequence identity were built using Uclust [11]. Then the non-redundant dataset was clustered to the superfamily level using the profile-profile comparison method HHsearch [12] and the superfamily assignment was checked by setting a minimum fold similarity probability thanks to the structural alignment of the structures using MATRAS, which combines secondary and 3D structure alignments [13,14]. Finally, structural interologs below 70% overall sequence identity were defined as pairs of interfaces AB and A'B', where A and B, A' and B' were clustered together at the superfamily and fold levels. It was checked that the positions involved in the interface of A and B (defined as the set of residues whose solvent-accessible surface area varies upon binding) overlapped with more than 40 % of the positions involved in the interface of A' and B', respectively. In large complexes such as the 20S proteasome where several domains from the same superfamily interact in different ways, we wanted to avoid detecting many pairs of interologs whose structures can diverge: thus, if two interfaces I1 and I2 had no structural similarity, but if, in the same pdb complex as I2, another interface I3 was similar to I1, the I1-I2 pair was not considered in the interolog detection.

For each pair of structural interologs, the interface root-mean square deviation (iRMSD) was re-computed using the same formula as in [10]. Briefly, iRMSDs were computed following a generalization of the iRMSD calculation as performed in the CAPRI contest [15,16]. Given a pair of complexes chainA-chainB and chainA'-chainB', chains A and A' were superimposed using MATRAS [13,14] and "common interface residues for A and A' " were defined as all the pairwise aligned positions involved in the interface of both AB and A'B' complexes, respectively. The same calculation was repeated for chains B and B' to define the "common interface residues for B and B' ". Two interface iRMSDs were computed using the coordinates of the backbone atoms: first, chains A and A' were aligned and an iRMSD_BB' was computed between common interface residues of chains B and B'; second, chains B and B' were aligned and an iRMSD_AA' was computed between the common interface residues of chain A and A'. The minimal value between both iRMSD_AA' and iRMSD_BB' was chosen as the representative iRMSD. Note that here the iRMSD results are slightly different from [10] because the residues involved in the interface are not exactly the same, as the interface was detected in a different manner (see main text Methods for the definition of the interface).

In this study, our goal was to have enough pairs of interologs to carry out quantitative analyses, but it was also necessary to have very clean and precise data. To this effect, first, the interologs were carefully examined. The dataset was refined by eliminating all groups including more than 10 structural interologs and groups containing interfaces from very large complexes such as hemoglobins, erythrocruorins, photosystems, cytochromes, ribosome, in order to avoid large biases in the analysis. If two interologs had one complex chain in common (same PDB id, same chain) then the pair was removed to avoid comparing different interfaces from the same complex. Structures determined using NMR or EM and structures containing single small domains or chains with very generic tertiary structures (such as coiled coils, e.g. SNAREs) were also removed from the dataset because of the need for structural accuracy down to the residue level and reliability of the structural alignment of interolog pairs. Finally, pairs of interologs with a high interface root-mean square deviation (over 8 Å), which could be identified as corresponding to different conformations or erroneous structural alignments, were removed.

The final interolog datasets of predicted non-obligate and obligate interfaces are composed of groups of structural interologs containing 1 to 27 interolog pairs. In order to be as confident as possible in the quality of the data, the whole analysis is based on interolog pairs, although interolog groups were used as a control to check that the heterogeneity in the number of pairs each group contains does not bias the results.

The interologs were sorted in two datasets based on the automated NOXclass prediction [3] of the non-obligate or obligate nature of the interaction. As an alternative, each group of interologs was also manually assigned a non-obligate or obligate character depending on the nature of the interfaces that it contained. 85% of the predictions are common between the NOXclass prediction and the manual curation.

Among the non-obligate complexes, we isolated a subset of 60 pairs that we confidently assigned to the class of transient interactions. This subset is not exhaustive. In particular, we kept a maximum of one pair of interologs per group, meaning that two pairs I1-I2 and I3-I4 from the transient subset do not contain mutually interologous complexes (I1-I3, I1-I4, I2-I3 and I2-I4 are not interolog pairs). Moreover, we eliminated receptors and strong interactions, such as protease inhibitors. The resulting transient subset is distributed over the whole range

of minimum interface sequence identity: 19 pairs with 0-30% identity, 16 pairs with 30-50%, 15 pairs with 50-70% and 10 pairs with over 70% identity. The corresponding pairs are identified in Dataset S1.

Every interolog pair was manually assigned an "orthologous" or "paralogous" character. Due to the difficulty of discriminating true orthologs and paralogs using phylogenetic criteria, especially considering the existence of in-paralogy and out-paralogy [17], this classification was performed empirically based on the organism to which each interface belongs and the function of the complex as described in the PDB entry. When the proteins involved were enzymes, the enzyme nomenclature (EC classification) accessible from the PDB entry was used. The corresponding datasets contain a mixture of predicted non-obligate and obligate interactions.

Finally, as a control of the influence of the crystallographic data quality on the analysis, a redundant, non-exhaustive dataset *redundant95* was built, containing pairs of complexes with at least 95% overall sequence identity between both pairs of chains. To this effect, the list of redundant interfaces at 70% overall sequence identity available from the InterEvol database website was used [10]. From each redundant group as many pairs of heteromeric interfaces as possible were extracted with a minimum of 95% overall sequence identity, with the diversity constraint that the same complex (as identified by its PDB accession number and chain names) must not appear twice in *redundant95*. 12 pairs with less than 70% identity at interface or an interface root-mean-square deviation over 7 Å were eliminated because they either correspond to different conformations or include disordered regions which are difficult to align structurally. 4 pairs including NMR structures were discarded. This left 387 pairs of redundant structures, of which 188 have a global sequence identity of 100% on both chains. Two subsets of *redundant95*, defined by restriction to X-ray structures with a resolution better than, respectively, 2.5 Å and 2.0 Å, contain respectively 86 pairs (dataset *redundant95res2.5*) and 17 pairs (dataset *redundant95res2.0*). The results for these redundant datasets are considered as an estimation of the variability introduced by local structural heterogeneity in the analysis: for the same complex, crystallized in different conditions, there can be variations in the positions of the side chains accounting for at least part of the non-conserved contacts [18,19].

The composition of each dataset is detailed in Dataset S1. The chain names do not necessarily match with those in the original PDB file because in the process of building the InterEvol database, some chains were renamed. The chain names in Dataset S1 thus correspond to those available in the InterEvol database.

The distribution of the interolog pairs in the main dataset according to the sequence identity at interface is represented in Supporting Figure 1A in Text S2. The quality of the interolog data is confirmed by the distribution of the minimum interface root-mean-square deviation (iRMSD) depending on sequence identity at interface in Supporting Figure 1B in Text S2 (one data point for each pair of interologs). The distribution of interface size (represented by the number of residues in each interface) is represented in Supporting Figure 1C in Text S2.

16. Definition of atomic contacts

Atomic contacts were calculated using an α-shape representation of the interface, based on a Laguerre polyhedral decomposition where the initial set of points is the set of atomic coordinates of surface atoms belonging to interface residues in both chains involved in the

interface [20]. The α-shape is a geometric representation providing a unique decomposition of the volume occupied by the atoms and thus giving the global shape of the object as well as the atomic contacts within the object (here, one chain or two chains forming an interface). A standard implementation of the method, using the van der Waals radius of each atom, gave the atomic interface contacts. Only the vertices connecting two atoms at the surface of each of the two interface sides (their atomic accessibility given by NACCESS [21] is different in the free and the bound state) and belonging to interface residues were selected. The method was implemented using CGAL [CGAL, Computational Geometry Algorithms Library, http://www.cgal.org]. We used a solvent parameter of 0.4 (meaning that we add 0.4 Å to the radius of each atom) and an alpha parameter of 0.0. A manual inspection of some representative interfaces from the datasets showed that this definition was more accurate and relevant than a representation based purely on the distance between atoms, because it better depicts the micro-environment of interface atoms.

This gave a set of atomic contacts between surface atoms belonging to interface residues. In order to compare the contacts between pairs of interologs, these atomic contacts were translated into contacts between pairs of residues. Each contact between two residues (one in each partner) was characterized by the number of atomic contacts that could be identified between these two residues. Contact conservation was then calculated over all pairs of interface residues, as the sum of atomic contacts conserved between the same residue positions in the two interologs, divided by the sum of conserved and non-conserved atomic contacts (the non-conserved atomic contacts are contacts occurring between two residues in one interolog, while the corresponding residues in the other interolog are not in contact). This corresponds to the Jaccard index (similarity coefficient) between the graphs of contacts in each interolog, with weighted edges: for atomic contacts, if a contact between two positions exists in both interologs, the corresponding edge of the graph is weighted by the average number of atomic contacts between the two positions; if the contact exists in only one of the two interologs, the edge is weighted by the number of atomic contacts in the interolog where the contact exists.

17. Notion of geometric center of the interface, of an apolar patch

The geometric center was used as a reference center to account for plasticity at the contours of the interface or an apolar patch. The geometric center for each side of the interface was defined by extracting the Cα coordinates of each interface residue on this side and calculating the coordinates of their geometric center (barycenter). The distance from each residue to this geometric center was then calculated as the distance from the residue's Cα to the geometric center. There is thus one geometric center on each side of the interface. The central residue of each side of the interface was defined as the closest residue to the geometric center.

Similarly, for each apolar patch, a geometric center was defined based on the Cα coordinates of the residues in this patch. The distance from each residue in the patch to the geometric center of the patch was then calculated.

18. Definition of apolar contacts and apolar patches

In order to investigate the conservation of apolar contacts, two definitions were used. Both definitions are based on the α-shape representation of atomic contacts rather than the basic distance definition used to identify the interface residues, because hydrophobicity involves contacts at longer distances and is thus more sensitive to the way contacts are described.

The first definition relied on a very simple and restrictive description of "apolarity": the initial set of points for the α-shape was limited to the apolar surface interface atoms in amino acids usually considered hydrophobic, namely Met, Val, Leu, Ile, Phe, Trp, Tyr [22,23]. The second definition was based on previous work on hydrophobic patches [24,25] and included all carbon and sulfur atoms from any type of amino acid provided that they were surface interface atoms belonging to interface residues. Apolar contacts were calculated using the α-shape representation of atomic contacts, including all C and S surface interface atoms belonging to interface residues and using a van der Waals radius expansion of 0.7 Å (half the standard probe size) for polar atoms.

Building up on the second definition, apolar patches were also defined for each side of the interface based on the α-shape connections between surface interface atoms. The apolar patches were identified using a standard clustering method. To avoid narrow strings of apolar atoms which artificially connect two patches [25], an expansion of the van der Waals radii of polar atoms (N and O) by a fixed amount of 1.4 Å (corresponding to the standard probe size) was applied. This definition leads to more specific patches compared to other values of the polar expansion. Apolar patches containing atoms from the same residue were then merged because comparison between interologs is based on a structural alignment at the residue level. In order to fully enable comparison between patches from a pair of interologs, apolar patches were also iteratively merged so that one patch in one interolog corresponded to maximum one patch in the other interolog (when it corresponded to two patches, those two patches were fused). Patches that corresponded to each other between interologs were termed equivalent. Two patches (one on each side of the interface) were considered in contact if there was at least one apolar contact between two residues, one in each patch.

The construction of apolar patches is illustrated in Supporting Figure 7A in Text S2.

19. Multiple sequence alignments and evolutionary rate of interface residues

The generation of multiple sequence alignments was restricted to interfaces where both chains came from species belonging to the same "domain of life": bacteria, archaea or eukarya [26]. This brought the total number of interfaces down from 965 to 889. A series of pairs of multiple sequence alignments was generated for those interfaces. The method used was InterEvolAlign with its standard options, described in [10]. This method produced two multiple sequence alignments (one for each interface chain) containing most likely orthologous sequences from the same ordered species. This protocol limits the inclusion of false orthologs in the alignments. The series of alignments was generated using 2 iterations over the "Entire genomes (OMA)" database [27] and an additional iteration over the NCBI RefSeq database [28]. The number of common species retrieved was limited to 100 in order to speed up the process and enable calculations of evolutionary rates over a reasonable timelength.

Out of 1024 interolog pairs, 201 could not be compared using these multiple sequence alignments because no alignment could be generated for at least one of the two interologs: this corresponds to cases of hybrid interfaces, cases where no sequence could be retrieved for one

of the two chains at least and cases where there was no common species between the two alignments. Among the pairs that could be compared, 86% of the multiple sequence alignments both contained more than 10 sequences. There were on average 53 sequences per multiple sequence alignment and 31 common species between the alignments of the two interologs.

We checked that the conservation profile of the interface corresponded to what we expected in terms of residue burial [29,30]. The core residues are significantly more conserved than average and the support residues are even more conserved, whereas the rim region evolves more quickly (p-value < 2.2e-16 for any two sub-regions and for any sub-region vs. all residues).

Also, evolutionary rate estimates are coherent between interologs. Evolutionary rate estimates computed using Rate4Site can be compared between each pair of structurally equivalent interface residues belonging to a pair of interologs. The calculation of evolutionary rate estimates between the two interologs had to be consistent because the rates were used to reconsider contact conservation. The analysis was restricted to evolutionary rates calculated from multiple sequence alignments containing more than 10 sequences. The correlation coefficients between the distributions of evolutionary rate estimates in two interologs are 0.55 for 0-30% minimum interface sequence identity, 0.78 for 30-50%, 0.87 for 50-70% and 0.92 for 70-100%. In particular, for the normalized rates above 80 that were typically used, there is a good coherence in evolutionary rates at interface in each pair of interologs.

20. Statistical analysis, logistic regression and graphics

The R package was used to perform statistical tests and draw the boxplots [31].

In the boxplots, the bold line represents the median, boxes enclose the first and third quartiles of the distribution and whiskers extend to the most extreme data point which is no more than 1.5 times the interquartile range from the box.

All p-values presented in the main text of the paper as well as in the supporting material were calculated using non-parametric Wilcoxon rank sum tests.

As an alternative to non-parametric Wilcoxon rank sum tests, comparisons with Welch two sample t-tests were tested and yielded very similar results with no change in the conclusions about statistical significance.

The confidence intervals (displayed as "error bars") in main Figure 2 and Supporting Figures 7D and 8F were obtained by performing a bootstrap on the population of 1,024 interolog couples. This consisted in randomly drawing one half of the dataset (without replacement) one thousand times, calculating the mean value of contact conservation in each of the 1,000 resampled populations and extracting the intervals containing 95% of the calculated mean values.

The logistic regression models were built and analyzed using the R package [31].

References

1. Chakrabarti P, Janin J (2002) Dissecting protein-protein recognition sites. Proteins 47: 334-343.
2. Bahadur RP, Chakrabarti P, Rodier F, Janin J (2004) A dissection of specific and non-specific protein-protein interfaces. J Mol Biol 336: 943-955.
3. Zhu H, Domingues FS, Sommer I, Lengauer T (2006) NOXclass: prediction of protein-protein interaction types. BMC Bioinformatics 7: 27.
4. Kabsch W, Sander C (1983) Dictionary of protein secondary structure: pattern recognition of hydrogen-bonded and geometrical features. Biopolymers 22: 2577-2637.
5. Levy ED (2010) A simple definition of structural regions in proteins and its use in analyzing interface evolution. J Mol Biol 403: 660-670.
6. McDonald IK, Thornton JM (1994) Satisfying hydrogen bonding potential in proteins. J Mol Biol 238: 777-793.
7. Rajamani D, Thiel S, Vajda S, Camacho CJ (2004) Anchor residues in protein-protein interactions. Proc Natl Acad Sci U S A 101: 11287-11292.
8. Meireles LM, Domling AS, Camacho CJ (2010) ANCHOR: a web server and database for analysis of protein-protein interaction binding pockets for drug discovery. Nucleic Acids Res 38: W407-411.
9. Dalgaard P (2008) Logistic regression. In: Springer, editor. Introductory Statistics With R. pp. 227-248.
10. Faure G, Andreani J, Guerois R (2012) InterEvol database: exploring the structure and evolution of protein complex interfaces. Nucleic Acids Res 40: D847-856.
11. Edgar RC (2010) Search and clustering orders of magnitude faster than BLAST. Bioinformatics 26: 2460-2461.
12. Soding J (2005) Protein homology detection by HMM-HMM comparison. Bioinformatics 21: 951-960.
13. Kawabata T, Nishikawa K (2000) Protein structure comparison using the markov transition model of evolution. Proteins 41: 108-122.
14. Kawabata T (2003) MATRAS: A program for protein 3D structure comparison. Nucleic Acids Res 31: 3367-3369.
15. Janin J, Henrick K, Moult J, Eyck LT, Sternberg MJ, et al. (2003) CAPRI: a Critical Assessment of PRedicted Interactions. Proteins 52: 2-9.
16. Mendez R, Leplae R, De Maria L, Wodak SJ (2003) Assessment of blind predictions of protein-protein interactions: current status of docking methods. Proteins 52: 51-67.
17. Sonnhammer EL, Koonin EV (2002) Orthology, paralogy and proposed classification for paralog subtypes. Trends Genet 18: 619-620.
18. Best RB, Lindorff-Larsen K, DePristo MA, Vendruscolo M (2006) Relation between native ensembles and experimental structures of proteins. Proc Natl Acad Sci U S A 103: 10901-10906.
19. Perica T, Chothia C (2010) Ubiquitin--molecular mechanisms for recognition of different structures. Curr Opin Struct Biol 20: 367-376.
20. Poupon A (2004) Voronoi and Voronoi-related tessellations in studies of protein structure and interaction. Curr Opin Struct Biol 14: 233-241.
21. Hubbard SJ, Thornton JM (1993) NACCESS.
22. Rose GD, Wolfenden R (1993) Hydrogen bonding, hydrophobicity, packing, and protein folding. Annu Rev Biophys Biomol Struct 22: 381-415.
23. Janin J (1979) Surface and inside volumes in globular proteins. Nature 277: 491-492.
24. Lijnzaad P, Berendsen HJ, Argos P (1996) A method for detecting hydrophobic patches on protein surfaces. Proteins 26: 192-203.
25. Lijnzaad P, Argos P (1997) Hydrophobic patches on protein subunit interfaces: characteristics and prediction. Proteins 28: 333-343.

26. Woese CR, Kandler O, Wheelis ML (1990) Towards a natural system of organisms: proposal for the domains Archaea, Bacteria, and Eucarya. Proc Natl Acad Sci U S A 87: 4576-4579.
27. Altenhoff AM, Schneider A, Gonnet GH, Dessimoz C (2011) OMA 2011: orthology inference among 1000 complete genomes. Nucleic Acids Res 39: D289-294.
28. Pruitt KD, Tatusova T, Klimke W, Maglott DR (2009) NCBI Reference Sequences: current status, policy and new initiatives. Nucleic Acids Res 37: D32-36.
29. Eames M, Kortemme T (2007) Structural mapping of protein interactions reveals differences in evolutionary pressures correlated to mRNA level and protein abundance. Structure 15: 1442-1451.
30. Franzosa EA, Xia Y (2009) Structural determinants of protein evolution are context-sensitive at the residue level. Mol Biol Evol 26: 2387-2395.
31. Ihaka R, Gentleman R (1996) R: A language for data analysis and graphics. Journal of Computational and Graphical Statistics 5: 299-314.
