# Supplementary material for: Versatility and Invariance in the Evolution of Homologous Heteromeric Interfaces
Source: PLoS Comput Biol. 2012 Aug 30;8(8):e1002677. doi: 10.1371/journal.pcbi.1002677 (PMC3431345; doi:10.1371/journal.pcbi.1002677)
Supplement: Text S2 — Supplementary tables (4 tables) and figures (8 figures). (PDF) [file pcbi.1002677.s003.pdf]

Text S2 – Supplementary tables and figures

Supporting Table 1. Abundance of whole interface (I), core (C), support (S) and rim (R) residues (as measured by the number of residues or the area contributed to the interface) in all interfaces present in the dataset of interologs and in the subsets of non-obligate and obligate interfaces. Comparison with the values obtained from a dataset of 70 complexes by Chakrabarti & Janin (Proteins 2002) [CJ02].

	All interfaces (956)								Non-obligate interfaces (544)								Obligate interfaces (412)								70 interfaces from [CJ02]					
	Number				Area				Number				Area				Number				Area				Number			Area		
	I	C	S	R	I	C	S	R	I	C	S	R	I	C	S	R	I	C	S	R	I	C	S	R	I	C	R	I	C	R
A	6.4	7.2	8.5	4.9	4.1	4.9	4.3	3.0	5.8	6.5	8.1	4.2	3.8	4.7	4.4	2.6	7.0	7.8	8.8	5.5	4.3	5.1	4.2	3.3	3.9	4.0	3.8	2.8	2.7	3.1
C	1.9	1.9	4.2	0.8	1.1	1.4	2.5	0.5	2.4	2.4	5.6	1.0	1.3	1.5	3.0	0.6	1.4	1.5	2.9	0.6	1.0	1.2	2.1	0.5	3.5	4.7	2.1	1.7	1.9	1.3
D	5.9	4.4	4.1	7.8	4.6	3.8	3.0	5.9	6.2	4.8	4.6	7.9	4.8	4.2	2.8	6.0	5.6	4.1	3.6	7.6	4.4	3.5	3.1	5.9	6.6	5.4	8.0	5.1	4.5	6.6
E	6.6	4.5	3.6	9.5	6.4	4.8	4.2	9.0	7.0	4.7	3.9	10.0	7.1	5.1	4.9	9.8	6.2	4.3	3.4	9.1	5.9	4.5	3.6	8.3	6.5	4.6	8.6	6.0	4.4	10.0
F	4.5	5.2	6.0	3.4	6.0	6.5	8.1	4.9	3.7	4.1	4.9	2.8	4.7	4.9	6.6	4.0	5.3	6.1	6.9	3.9	7.1	7.8	9.3	5.8	3.5	5.1	1.7	4.1	5.5	1.1
G	7.0	7.7	8.6	5.6	3.5	4.0	4.3	2.6	7.0	8.3	8.3	5.6	3.6	4.4	4.8	2.5	6.9	7.2	8.9	5.6	3.3	3.7	3.8	2.7	8.1	7.5	8.7	4.8	4.2	6.4
H	2.7	2.5	3.3	2.4	2.9	2.9	4.3	2.5	2.8	2.4	4.1	2.5	2.9	2.7	5.4	2.6	2.5	2.6	2.7	2.4	2.8	3.0	3.4	2.5	3.4	4.4	2.3	3.8	4.4	2.4
I	5.0	5.6	6.1	4.1	5.4	6.0	5.2	4.6	4.6	5.2	5.7	3.8	4.7	5.4	4.6	4.0	5.4	6.0	6.5	4.4	5.9	6.5	5.8	5.1	3.6	4.1	3.1	4.6	4.9	3.5
K	5.3	3.4	2.6	7.9	5.7	4.0	3.8	8.2	5.7	3.7	2.6	8.4	6.3	4.5	3.6	9.0	4.9	3.2	2.5	7.3	5.2	3.6	3.9	7.6	5.7	3.7	8.0	6.5	5.2	9.7
L	8.3	9.3	9.4	7.0	10.0	11.0	9.7	8.8	7.4	8.8	9.0	5.8	9.1	10.7	9.5	7.2	9.0	9.7	9.9	8.0	10.7	11.3	10.0	10.2	5.0	5.5	4.5	5.7	5.8	5.3
M	2.6	3.0	2.7	2.3	3.2	3.4	3.3	2.9	2.2	2.3	2.1	2.2	2.6	2.6	2.2	2.8	3.0	3.5	3.3	2.4	3.7	4.1	4.1	3.1	2.0	2.6	1.4	3.2	3.7	2.0
N	4.1	4.2	3.0	4.6	3.9	3.9	2.9	4.3	4.5	4.5	3.0	5.1	4.4	4.2	2.9	4.9	3.8	4.0	3.0	4.1	3.6	3.7	2.8	3.7	5.9	5.4	6.4	5.7	5.4	6.4
P	5.0	5.2	3.5	5.7	4.6	4.7	3.1	4.9	4.6	4.6	2.5	5.6	4.1	4.2	2.0	4.6	5.5	5.6	4.4	5.8	5.0	5.1	4.0	5.1	3.8	3.4	4.2	3.6	3.5	4.1
Q	4.0	4.1	2.8	4.4	4.7	4.7	4.2	4.9	4.5	4.6	3.0	5.1	5.5	5.5	4.5	5.8	3.5	3.7	2.6	3.9	4.1	4.1	3.9	4.2	3.7	3.7	3.8	4.3	4.3	4.2
R	6.8	6.1	4.6	8.4	10.4	9.0	9.2	12.5	7.0	6.1	4.8	8.7	11.0	9.4	9.6	13.3	6.6	6.1	4.4	8.1	9.9	8.7	8.8	11.8	6.4	5.9	7.0	10.1	10.1	9.9
S	6.1	6.4	5.8	5.9	4.0	4.4	3.6	3.7	6.6	7.0	6.7	6.2	4.4	4.9	4.3	3.9	5.6	5.9	5.0	5.7	3.7	4.1	3.0	3.5	7.9	7.8	8.1	5.4	4.8	7.3
T	5.6	6.1	4.9	5.6	4.7	5.1	3.4	4.4	6.0	6.8	4.9	6.0	5.1	5.6	3.5	4.9	5.3	5.6	5.0	5.2	4.3	4.7	3.3	4.0	6.2	5.7	6.8	5.0	4.7	5.9
V	5.9	6.7	7.4	4.7	5.4	6.1	5.7	4.4	5.6	6.5	7.3	4.2	5.1	6.0	5.6	4.0	6.2	6.9	7.5	5.1	5.6	6.3	5.7	4.8	4.5	4.3	4.7	3.8	3.8	3.9
W	1.8	1.8	2.9	1.3	2.8	2.8	5.5	2.2	1.8	1.7	3.2	1.3	2.9	2.8	6.9	2.0	1.8	1.8	2.6	1.4	2.8	2.8	4.4	2.4	2.8	4.1	1.3	4.2	5.3	1.6
Y	4.6	4.7	6.1	3.8	6.6	6.7	10.0	5.8	4.6	5.0	5.8	3.7	6.6	6.9	9.1	5.7	4.6	4.5	6.4	3.8	6.7	6.5	10.8	5.9	6.8	8.1	5.4	9.4	10.9	5.3

Supporting Table 2. Details of the logistic regression models for the switching out predictor and the contact conservation predictor. The deviance from LRT (Likelihood Ratio Test) was obtained by dropping each parameter one at a time and assessing the corresponding variation in deviance. The deviance from the progressive inclusion of all 6 parameters (in the order determined by their rank, obtained from the previous "dropping" analysis) is also reported. The significance (Signif.) of each parameter was assessed both from the z-tests performed on the logistic regression coefficients and from the deviance test: significance values were always found to be < 2.2e-16 (indicated by *** in the table). Standard deviations (sd) are reported over 10 repeats.

		Switching out predictor					Contact conservation predictor				
**Category**	**Features**	Coefficient in logistic regression equation	Signif.	Rank in deviance reduction analysis	Variation in deviance (LRT, "dropping")	Variation in deviance (progressive inclusion)	Coefficient in logistic regression equation	Signif.	Rank in deviance reduction analysis	Variation in deviance (LRT, "dropping")	Variation in deviance (progressive inclusion)
Sequence Features	Similarity residue (BLOSUM62)	-0.054 (sd 0.005)	***	6	115 (sd 21)	115 (sd 21)	0.112 (sd 0.005)	***	3	858 (sd 102)	1688 (sd 192)
	Similarity environment (BLOSUM62)	-0.128 (sd 0.005)	***	3	513 (sd 50)	742 (sd 74)	0.224 (sd 0.007)	***	1	3033 (sd 207)	4638 (sd 423)
	Overall sequence id	-0.0096 (sd 0.0010)	***	5	220 (sd 49)	509 (sd 74)	0.0066 (sd 0.0009)	***	5	162 (sd 43)	156 (sd 39)
Geometric Features	Core / Support / Rim	0 (sd NA) / 0.96 (sd 0.08) / 1.06 (sd 0.07)	***	2	566 (sd 77)	920 (sd 97)	0 (sd NA) / 0.23 (sd 0.04) / -0.21 (sd 0.03)	***	4	191 (sd 29)	316 (sd 38)
	Number of atomic contacts per residue	-0.122 (sd 0.006)	***	1	1381 (sd 88)	3585 (sd 143)	0.075 (sd 0.002)	***	2	1744 (sd 138)	2179 (sd 195)
	Distance to interface geometric center	1.40 (sd 0.09)	***	4	357 (sd 51)	371 (sd 55)	-0.69 (sd 0.06)	***	6	131 (sd 21)	131 (sd 21)
Intercept		-1.99 (sd 0.09)					-1.32 (sd 0.06)				

Supporting Table 3. List of 52 cases where for a given complex, the structures of both at least one interolog and a redundant complex (over 95% sequence identity) are available. When several interologous structures are available, they are indicated in the third column of the table. When several complexes from the same group of interologs are present in the table, this is indicated in the fourth column of the table (this corresponds to one case with 3 interologs belonging to the same group and 6 cases with 2 interologs belonging to the same group).

Complex of interest	Redundant complex	Interolog(s)	Interolog group
1twf_BA	3hou_BA	3h0g_BA, 2waq_BA, 2a6h_CD	
2a6h_CD	3aoh_CD	3h0g_BA, 2waq_BA, 1twf_BA	same as 1twf_BA
2waq_BA	2pmz_CA	3h0g_BA, 1twf_BA, 2a6h_CD	same as 1twf_BA
1y8q_BA	3kyc_BA	1tt5_BA	
1tt5_BA	3dbh_BA	1y8q_BA	same as 1y8q_BA
1twf_BJ	3hou_BL	2waq_BK, 3h0g_BL	
2waq_BK	2pmz_CK	1twf_BJ, 3h0g_BL	same as 1twf_BJ
2waq_FA	2pmz_HA	3h0g_CA, 1twf_EA	
1twf_EA	3hou_FA	3h0g_CA, 2waq_FA	same as 2waq_FA
1xwd_AB	1fl7_AB	1hcn_AB	
1hcn_AB	1qfw_AB	1xwd_AB	same as 1xwd_AB
2pa8_AB	2pmz_DI	1twf_CI	
1twf_CI	3gtm_CI	2pa8_AB	same as 2pa8_AB
1tgz_BA	2io2_HD	2bkr_BA, 2ckh_BA, 2iy1_BA, 2io0_BA, 1euv_BA	
2iy1_BA	2iy0_BA	2bkr_BA, 2ckh_BA, 2io0_BA, 1euv_BA, 1tgz_BA	same as 1tgz_BA
2jdi_EG	1h8e_EG	3oee_EG	
2h62_BC	1rew_BD	3evs_BC	
1gp2_CB	2bcj_CB	1got_CB	
2gc4_BC	2j56_BA	3c75_EF	
1ryp_WP	1fnt_WP	1q5q_NG, 3mi0_JI, 1ryp_HA	
3cx5_ML	1ezv_ML	1zrt_ED, 1bcc_DC, 2qjy_ED	
1jwi_BA	1uex_BA	1fvu_DC	
1rzh_BC	1l9b_BC	2wjn_DB	
1twf_BC	3hou_BC	2waq_BI, 3h0g_BE	
2jdi_FG	2v7q_JD	3oee_FG	
1fqj_AB	1fqk_AB	2ode_AB, 2gtp_AB, 2ihb_AB, 1agr_AB, 2v4z_AB	
2gsm_BA	1m56_BA	1fft_BA, 3hb3_BA, 1xme_BA, 1v54_BA	
3cx5_JL	1ezv_JL	1bcc_AC	
2jdi_DG	1h8e_DG	3oee_DG	
1tvk_AB	1sa0_CD	2btq_DB	
1dce_BA	1ltx_BA	3dra_BA, 1n4q_BA, 2h6f_BA	
1jat_BA	2gmi_BA	1j7d_AB	
Complex of interest	Redundant complex	Interolog(s)	Interolog group

1twf_FA	3hou_HA	2waq_DA, 3h0g_HA	
2cch_AB	1jsu_AB	1f5q_AB, 2jgz_AB, 2f2c_BA, 3mi9_AB, 1w98_AB	
1m1n_AB	2afh_AB	1qgu_CD, 2xdq_AB, 3aek_AB, 1mio_AB	
2h62_CA	1rew_CB	3evs_DB	
3cx5_KJ	1ezv_KJ	1hr6_AB, 1bcc_BA	
2r6g_AE	3fh6_CB	3dhw_DB, 2r6g_BD, 2onk_AC, 3d31_AC	
1q16_AB	1r27_AB	2ivf_AB	
3hou_FG	1y1v_FG	3h0g_CK	
2pu9_CA	2pvo_CA	2puk_BA	
1m1n_CB	2afh_AD	1qgu_CB, 3aek_AD, 1mio_CB	
2a6h_CA	3aoh_CA	2waq_BI	
3ge3_BF	3dhg_AC	2inc_BF	
1f45_BA	3hmx_BA	1p9m_DF	
1twf_AD	3hou_AE	3h0g_AG	
1twf_CJ	3hou_CL	2waq_IK, 3h0g_EL	
1twf_CH	3gtm_CH	2waq_IJ, 3h0g_ED	
1h8e_BG	2v7q_GD	3oee_BG	
1twf_BG	3hou_BI	3h0g_BI	
1twf_GA	3hou_IA	3h0g_IA	
1rzh_CA	1l9b_CA	1eys_DB, 2wjn_BC	

Supporting Table 4. Properties (abundance and conservation) of interface residues, anchor residues and residues in apolar patches in terms of amino acid type and sub-region of the interface.

Amino acid type or sub-region of the interface	Relative abundance at interface (%)	Relative abundance among anchor residues (%)	Relative abundance in apolar patches (weighted by contribution in number of atoms) (%)
A	6.4	1.1	3.3
C	1.9	0.1	1.9
D	5.9	1.6	1.3
E	6.6	3.5	2.8
F	4.5	9.9	13.3
G	7.0	0.2	0.6
H	2.7	2.2	2.2
I	5.0	6.2	8.2
K	5.3	4.9	4.0
L	8.3	14.5	14.2
M	2.6	3.4	4.5
N	4.1	3.2	1.0
P	5.0	3.3	5.0
Q	4.0	6.6	2.5
R	6.8	14.7	4.7
S	6.1	1.2	1.3
T	5.6	2.8	3.8
V	5.9	2.8	7.8
W	1.8	5.6	6.1
Y	4.6	12.3	11.6
Core	34	99	44
Support	24	1	27
Rim	42	0	29

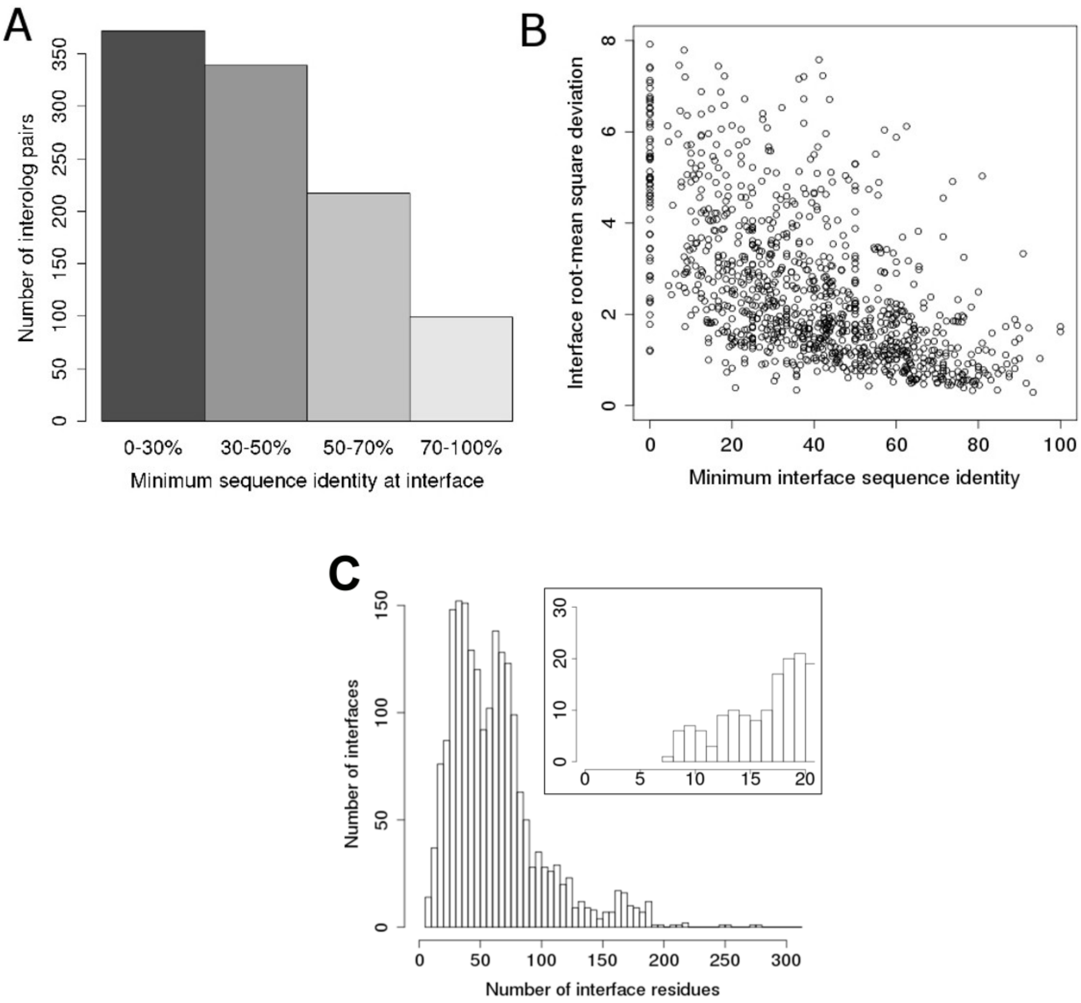

Supporting Figure 1. Properties of the 1,024 pairs of interologs in the whole interolog dataset (not including *redundant95*). (A) Distribution of the number of interolog pairs in the main dataset as a function of the minimum sequence identity at interface. (B) Distribution of the interface root-mean square deviation (iRMSD) of all pairs of interologs in the main dataset as a function of the minimum sequence identity at interface. (C) Distribution of interface size (expressed as the number of residues involved in each interface). In this distribution, each interface is counted once for each pair of interologs it is involved in. Inset: close-up on the 0-20 residue range.

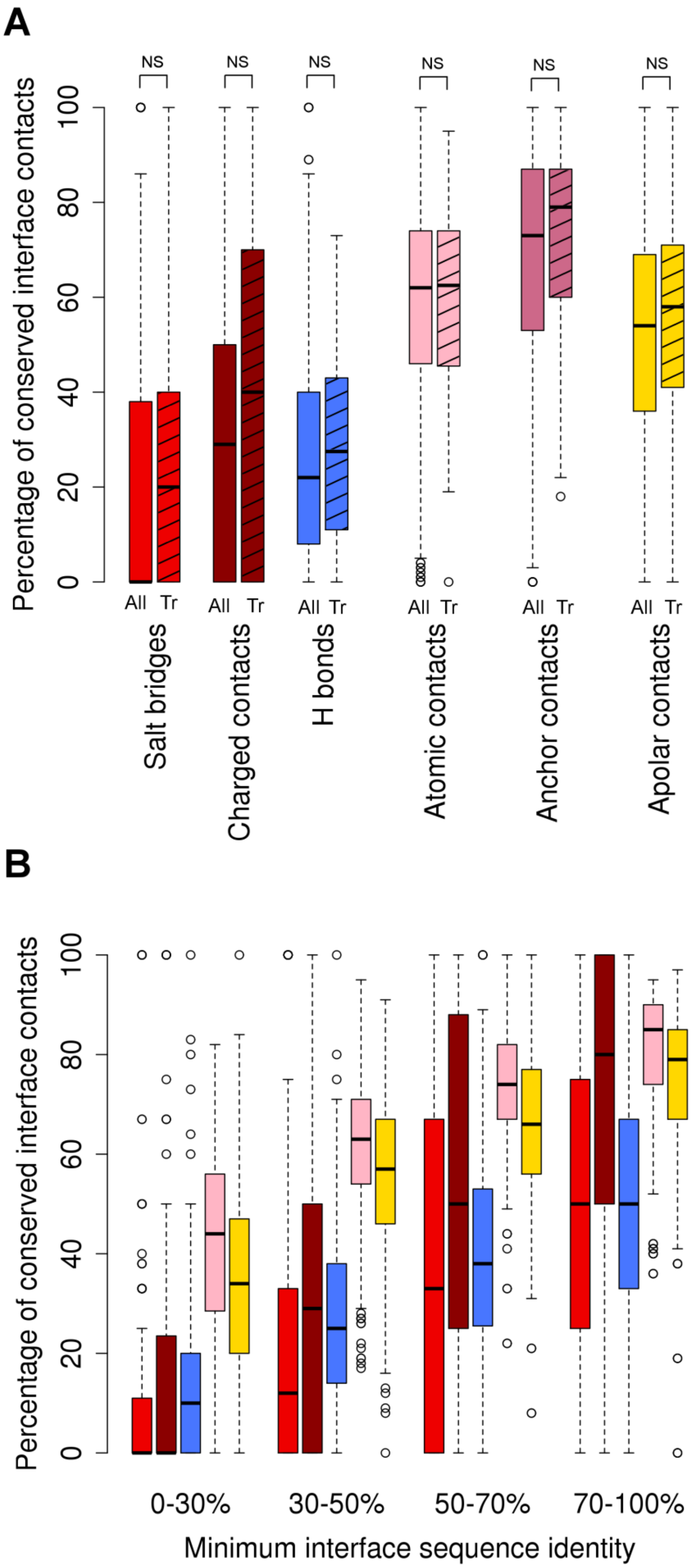
A
B
Percentage of conserved interface contacts
NS
All
Tr
Salt bridges
Charged contacts
H bonds
Atomic contacts
Anchor contacts
Apolar contacts
0-30%
30-50%
50-70%
70-100%
Minimum interface sequence identity

Supporting Figure 2. Distributions of the contact conservation values for all 1,024 pairs of interologs (All, not striped) and for a restricted subset of 60 transient interolog pairs (Tr for Transient, striped). From left to right, salt bridges (red), charged contacts at 5.5 Å (dark red), hydrogen bonds (blue), atomic contacts (pale pink), atomic contacts involving at least one anchor residue in either of the two interologs (dark pink), apolar contacts (gold). For any type of contacts, between the distribution for the whole dataset and the distribution for the 60 transient pairs, there is no significant difference (NS) (p-value > 0.1 in Wilcoxon rank sum tests). (B) Distributions of the conservation among different types of interface contacts. Colors are the same as in main Figure 2A. For all types of contacts, the differences between any two distributions of conservation among the four groups of sequence identities are statistically significant (p-value < 6.8e-3 in Wilcoxon rank sum tests).

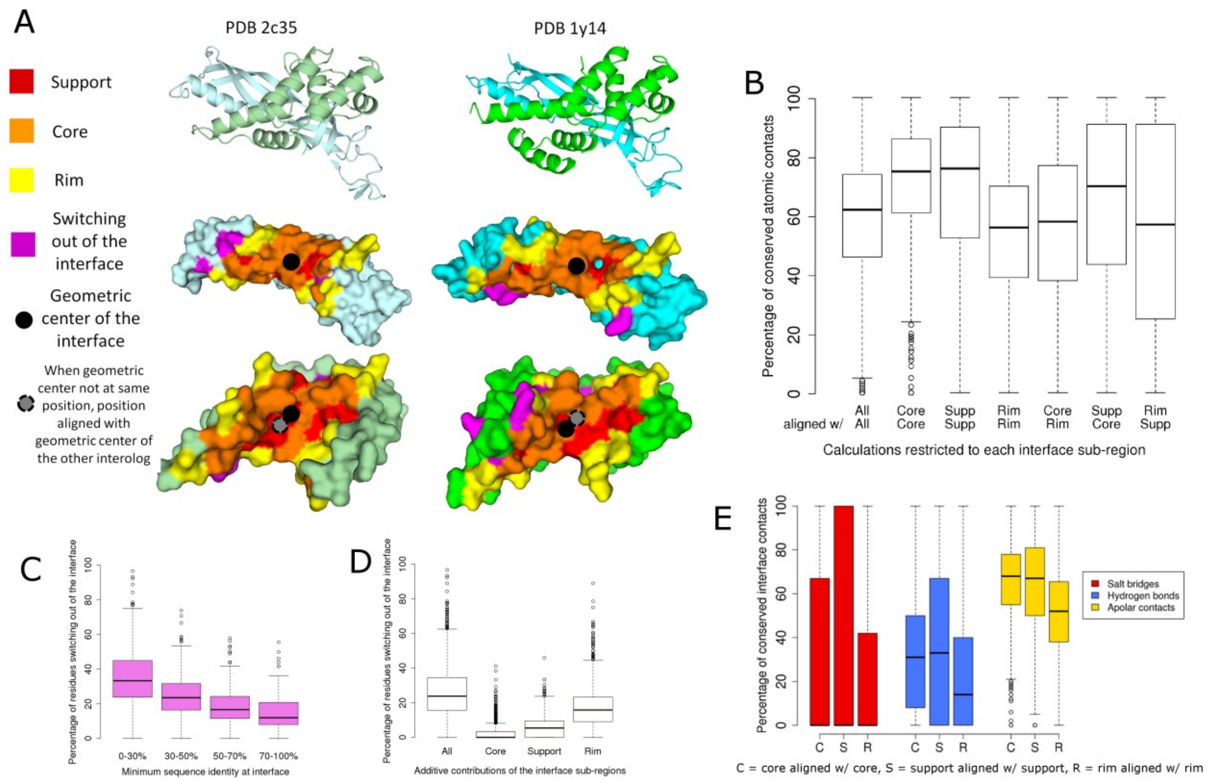

Supporting Figure 3. Core, support, rim and switching out of the interface. (A) Illustration of the different sub-regions of the interface, the residues switching out of the interface and the geometric center of the interface on a pair of interologs with 33% minimum interface sequence identity. (B) Distributions of the conservation of interface atomic contacts for residues split by interface sub-region: residues which are structurally aligned with a residue from the same sub-region of the interface (core aligned with core, support aligned with support or rim aligned with rim), then residues which are structurally aligned with a residue belonging to a different sub-region in the interolog. These distributions are compared to the global distribution for all atomic contacts (on the left, All aligned with All). The distributions for residues belonging to the core or the support in both interologs are all significantly higher than the distributions for residues belonging to the rim sub-region in at least one of the two interologs (all p-values in Wilcoxon rank-sum tests < 3e-5). (C-D) Switching out of the interface. Figure (C) shows the distribution of the proportion of residues switching out of the interface as a function of minimum sequence identity at interface. The differences between the four distributions (0-30%, 30-50%, 50-70%, 70-100%) are statistically significant (p-value in Wilcoxon rank sum tests < 6.6e-6). Figure (D) shows the additive contributions of the different interface sub-regions (core, support and rim) to the proportion of residues switching out of the interface. The core, support and rim distributions are significantly different from each other (p-value in Wilcoxon rank sum tests < 2.2e-16). (E) Percentage of conserved interface contacts including only residues which are structurally aligned with a residue from the same sub-region of the

interface (core aligned with core, support aligned with support or rim aligned with rim) for salt bridges (in red), hydrogen bonds (in blue) and apolar contacts (in golden yellow).

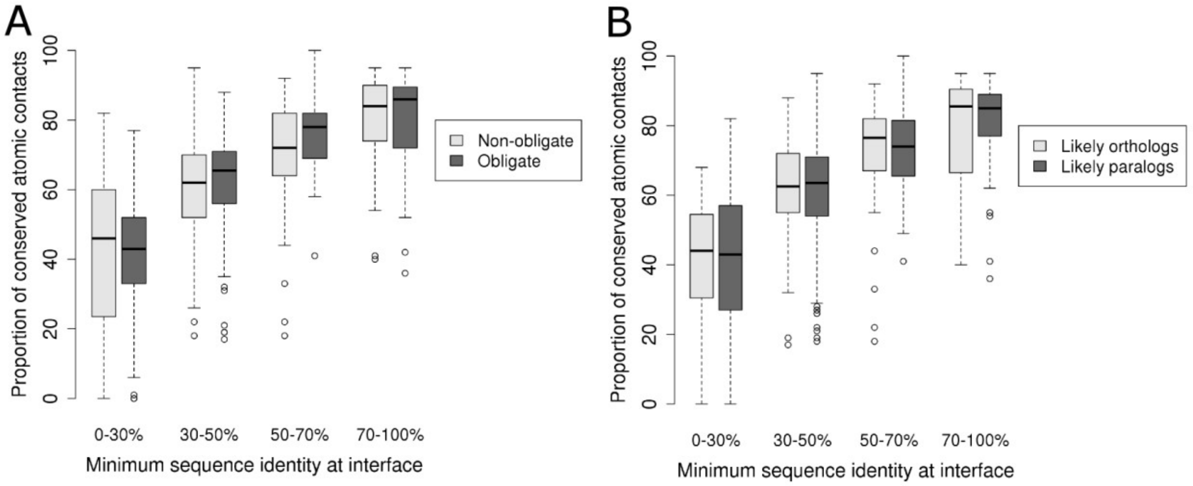

Supporting Figure 4. Influence of interaction characteristics on contact conservation. (A-B) Distributions of the proportions of conserved atomic contacts compared for predicted non-obligate interfaces (615 interolog pairs) and predicted obligate interfaces (409 interolog pairs) (A, light grey and dark grey) and likely orthologs (311 interolog pairs) and likely paralogs (713 interolog pairs) (B, light grey and dark grey). The differences for a given range of sequence identity are not significant.

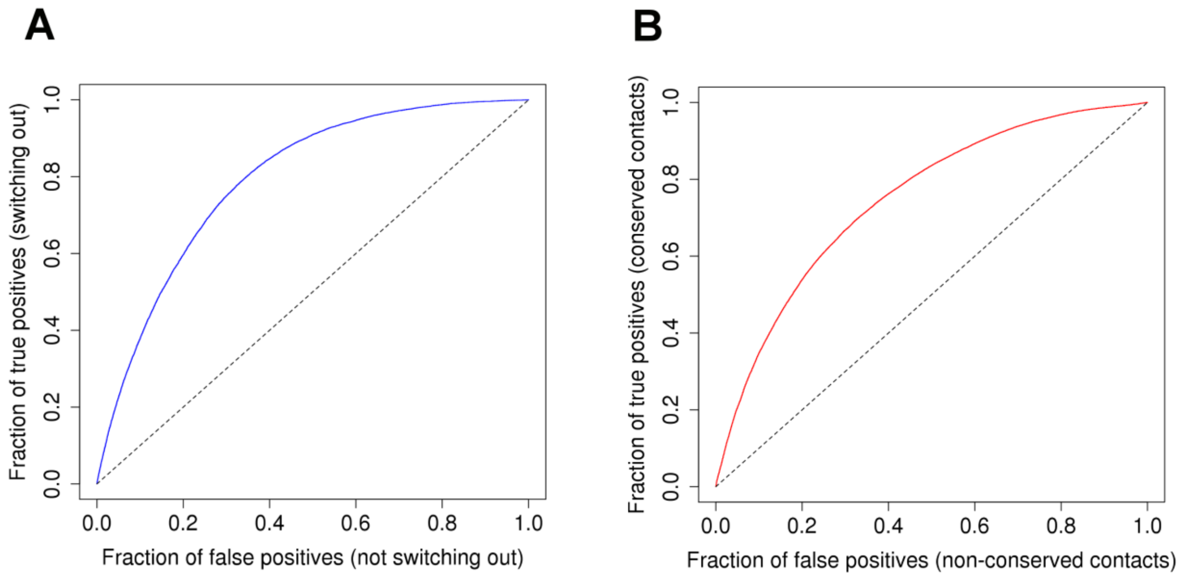

Supporting Figure 5. ROC curves for the two designed predictors. (A) ROC curve for the switching out predictor. This curve represents the fraction of true positives recovered (residues actually switching out of the interface) over the fraction of false positives recovered (residues not switching out of the interface) when integrating progressively all residues from all interfaces, sorted on the basis of their score in the switching out predictor (the higher the score, the higher the predicted probability to switch out of the interface). Area under the ROC curve (AUC): 0.79. (B) ROC curve for the atomic contact conservation predictor. This curve represents the fraction of true positives recovered (conserved contacts from all residues included so far) over the fraction of false positives recovered (non-conserved contacts, gained or lost by the residues included so far) when integrating progressively all residues from all interfaces, sorted on the basis of their score in the atomic contact conservation predictor (the higher the score, the higher the predicted probability for a residue both to conserve its contacts and not to gain or lose any contacts in the interolog). Area under the ROC curve (AUC): 0.75.

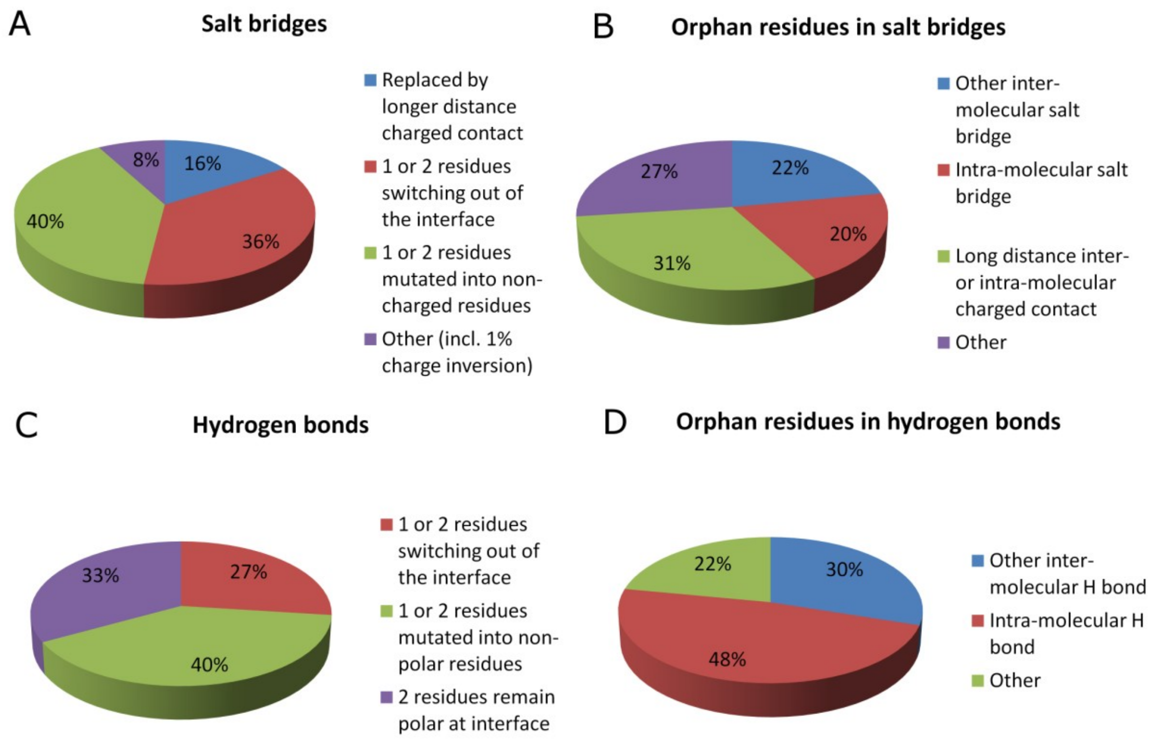

Supporting Figure 6. Mechanisms for the recovery of polar contacts. (A-B) Salt bridges. (A) Distribution of the cases of non-conserved salt bridges. (B) Scenarios of recovery for orphan residues (i.e. residues remaining charged but whose partner in one interolog switches out of the interface or is mutated into an uncharged residue). (C-D) Hydrogen bonds. (C) Distribution of the cases of non-conserved hydrogen bonds. (D) Scenarios of recovery for orphan residues (i.e. residues remaining polar but whose partner in one interolog switches out of the interface or is mutated into a non-polar residue).

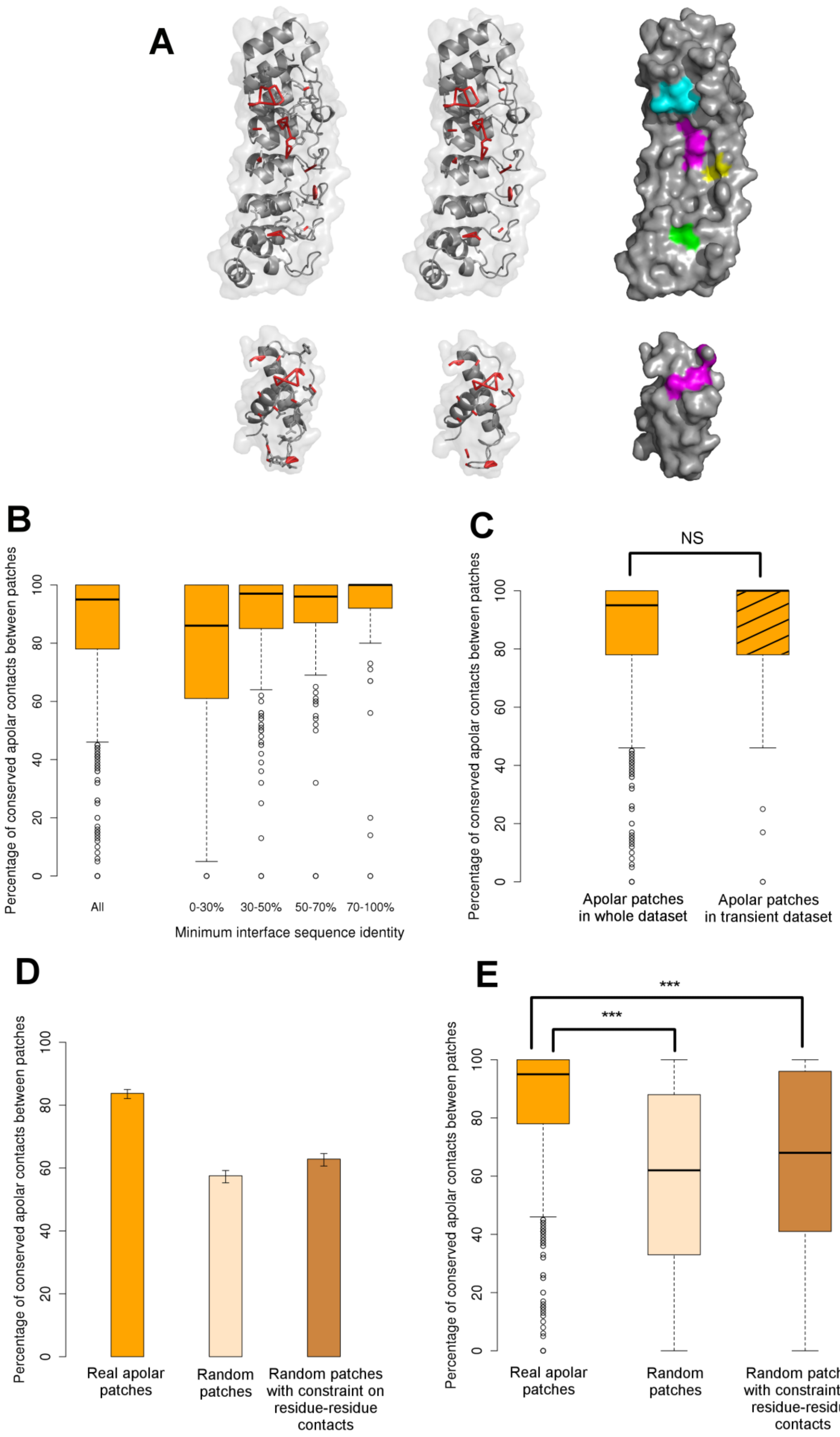
A
B
Percentage of conserved apolar contacts between patches
100
80
60
40
20
0
All
0-30%
30-50%
50-70%
70-100%
Minimum interface sequence identity
C
NS
Percentage of conserved apolar contacts between patches
Apolar patches in whole dataset
Apolar patches in transient dataset
D
Percentage of conserved apolar contacts between patches
Real apolar patches
Random patches
Random patches with constraint on residue-residue contacts
E
***
***
Percentage of conserved apolar contacts between patches
Real apolar patches
Random patches
Random patches with constraint on residue-residue contacts

Supporting Figure 7. Apolar patches. (A) Illustration of the construction of apolar patches on a complex between Nas6 and Rpt3 in yeast (PDB id 2dzn). On the left and in the middle, red cylinders depict apolar contacts between atoms on the surface of each chain (interface residues are represented as sticks on the left). On the right, apolar patches are colored on the surface of each chain. (B) Distribution of overall contact conservation ("All") and contact conservation depending on minimum interface sequence identity for apolar contacts between patches. There is a significant difference between 0-30% and 30-50% (p-value = 1.8e-10) and no significant difference between other sequence identity ranges (p-value > 0.05). (C) Distribution of contact conservation for apolar contacts between patches in the whole dataset (left, non-striped) and restricted to the dataset of 60 transient interolog couples (right, striped). The difference between the two distributions is non significant (NS) (p-value = 0.19 in a Wilcoxon rank-sum test). (D) Representation of the mean of each contact conservation distribution for apolar contacts between real apolar patches (left, orange), for apolar contacts between random patches (middle, beige) and for apolar contacts between random patches which were additionally constrained to have the same number of residue-residue contacts as the real apolar patches (right, light brown – see section 10 in Text S1 for a more detailed explanation). The confidence intervals contain 95% of the values obtained for the mean in a bootstrap procedure (1000 resamplings of half the distribution, without replacement). (E) Same as in (D), but with a boxplot representation of the contact conservation distributions. Contact conservation for both distribution concerning random patches is found to be significantly lower than the contact conservation for the real apolar patches (p-value < 2.2e-16 in Wilcoxon rank-sum tests).

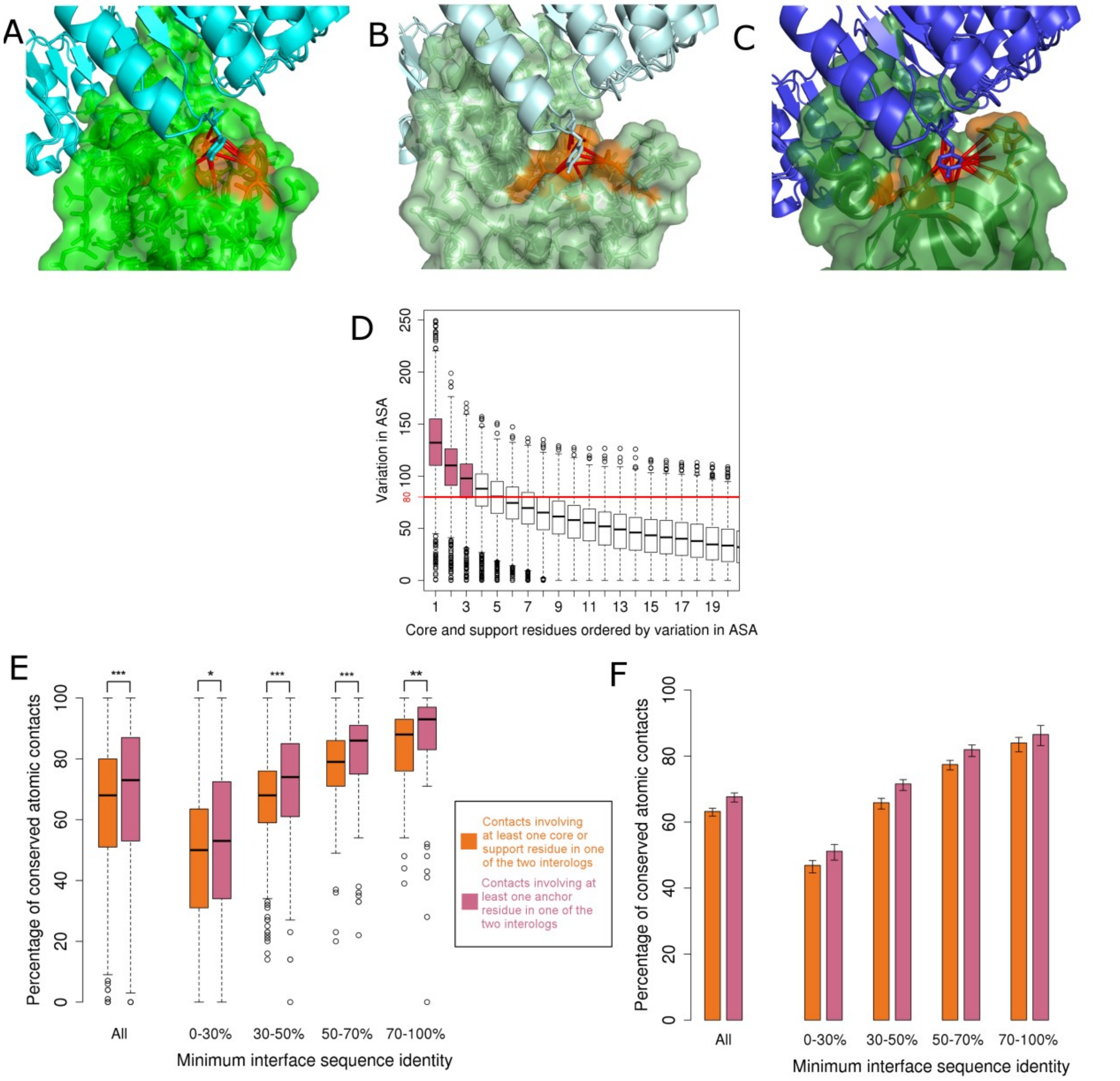

Supporting Figure 8. Anchor residues. (A-C) Illustration of the conservation of anchor residues and their contacts. The mutually interologous interfaces are three interfaces in Homo sapiens between a conserved partner (ribonuclease inhibitor, in cyan, light blue and dark blue) and three different partners with 30% to 38% mutual minimum interface sequence identity (in green, light green and dark green; respective PDB ids of the complexes 1a4y, 2bex, 1z7x). The row shows a conserved tyrosine in the blue chains which is an anchor in all 3 interfaces. This tyrosine makes atomic contacts with several residues (in orange) in the 3 interfaces; some of these residues are conserved in all 3 interfaces and others vary. Atomic contact conservation restricted to this residue ranges from 73% to 86% between the 3 pairs of interologs. (D) Distributions of the variation in ASA upon binding for the 20 core and support residues with the highest variation in each interface. Highlighted in pink are the 3 residues with largest variation which are chosen as anchor residues. The absolute 80 Å^2 threshold is

also displayed as a thick horizontal line. (E) Distribution of contact conservation overall ("All" = for all values of minimum sequence identity at interface) and depending on minimum interface sequence identity, for atomic contacts involving at least one residue from the support or core region in one of the two interologs (in reddish orange) and atomic contacts involving at least one anchor residue in one of the two interologs (in dark pink). The overall distributions ("All") between core/support contacts and anchor contacts are significantly different (p-value = 4e-8 in a Wilcoxon rank-sum test). The p-values in Wilcoxon rank-sum tests for 0-30%, 30-50%, 50-70% and 70-100% minimum interface sequence identity are respectively : 0.01, 9e-6, 7e-6 and 0.003. (F) Same as (E) but with the mode of representation used in main Figure 2: the mean of each distribution is represented as well as confidence intervals containing 95% of the values obtained for the mean in a bootstrap procedure (1000 resamplings of half the distribution, without replacement).
